# Rescue of tight junctional localization of a claudin-16 mutant D97S by antimalarial medicine primaquine in Madin-Darby canine kidney cells

**DOI:** 10.1038/s41598-019-46250-4

**Published:** 2019-07-04

**Authors:** Kana Marunaka, Naoko Fujii, Toru Kimura, Takumi Furuta, Hajime Hasegawa, Toshiyuki Matsunaga, Satoshi Endo, Akira Ikari

**Affiliations:** 1 Laboratory of Biochemistry, Department of Biopharmaceutical Sciences, Gifu Pharmaceutical University, Gifu, Japan; 2 Department of Pharmacology and Toxicology, Kyorin University School of Medicine, Tokyo, Japan; 3 Department of Pharmaceutical Chemistry, Kyoto Pharmaceutical University, Kyoto, Japan; 4 Saitama Medical Center, Saitama Medical University, Saitama, Japan

**Keywords:** Mechanisms of disease, Endocytosis, Adherens junctions, Protein translocation

## Abstract

Magnesium ion (Mg^2+^) is paracellularly reabsorbed through claudin-16 (CLDN16) in the thick ascending limb (TAL) of Henle’s loop in the kidney. Genetic disorders of CLDN16 cause mislocalization of CLDN16, resulting in hypomagnesemia. There is no effective treatment for hypomagnesemia except for magnesium administration. Here, we searched for a novel drug to restore tight junctional localization of a CLDN16 mutant. A D97S mutant, which has a mutation in the first extracellular loop (ECL) of CLDN16, was mainly colocalized with endosome marker, whereas wild-type (WT) CLDN16 was colocalized with ZO-1, an adaptor protein of tight junctions. The protein stability of the D97S mutant was lower than that of WT. The expression level of the D97S mutant was increased by lactacystin, a proteasomal inhibitor. Endocytosis inhibitors increased the tight junctional localization of the D97S mutant. We found that primaquine, an antimalarial agent, increased the protein stability and cell surface localization of the D97S mutant, but the localization of other mutants, which have mutations in the cytosolic domain or second ECL, was not affected. Transepithelial Mg^2+^ flux was increased by primaquine in D97S mutant-expressing cells. The expression of chaperon proteins, proteasome activity, and lactate dehydrogenase release were decreased by primaquine, and the proportion of viable cells increased. In contrast, these effects were not observed in WT CLDN16-expressing cells. These results suggested that primaquine increases the tight junctional localization of the D97S mutant, resulting in a reduction in ER stress and cellular injury. Primaquine may become an effective treatment drug for selected patients with mutant CLDN16.

## Introduction

Body Mg^2+^ content is rigorously regulated in the kidney. Approximately 80% of total plasma Mg^2+^ is daily filtered by the glomeruli followed by reabsorption from the renal tubules and only 5% is excreted into the urine^1,2^. The renal tubules are divided into several segments including the proximal tubule, thick ascending limb of Henle’s loop (TAL), and distal tubule, in which the reabsorption percentages are ~15%, ~60%, and ~5%, respectively. The transport properties of Mg^2+^ are different in each segment. The driving forces for the reabsorption of Mg^2+^ in the TAL are concentration gradient and transepithelial potential difference.

The paracellular permeability to ions in epithelial cells is controlled by intercellular junctions, especially tight junctions (TJs). The TJs barrier contains aqueous channels capable of discriminating charge and molecular size, and its electrical resistance varies among different epithelia^3,4^. Claudins (CLDNs) are tetraspan proteins with cytoplasmic amino and carboxyl termini, and comprise a large family of over 20 members^5,6^. In TJs, CLDNs can bind to scaffold proteins, including zonula occludens (ZO)-1 and ZO-2, mediated by the interaction bewteen a PDZ-binding motif and PDZ domains. ZO-1 and ZO-2 indirectly anchor CLDNs to the cytoskeletal network.

Familial hypomagnesemia with hypercalciuria and nephrocalcinosis (FHHNC) is characterized by hypomagnesemia, advanced nephrocalcinosis, and progressive renal failure^7^. The causative genes of FHHNC are *CLDN16* or *CLDN19*^8–10^. Both CLDNs can form homo- or hetero-oligomeric complexes and make divalent cation-permeable pores. Mg^2+^ may be paracellularly reabsorbed through the pores in the TAL^11^. CLDN14 interacts with CLDN16 to repress the cation selectivity of the CLDN16/CLDN19 complex, resulting in hypercalciuria^12^. Over 30 different mutations of CLDN16 are reported in FHHNC patients^13–16^. Recently, Sikora *et al*. reported that a missense variant of Leu151Phe is the most common mutation of FHHNC in Poland, but the percentage of each mutation in the World was unknown. Various mutants are mainly distributed into the cytosolic compartments including the Golgi apparatus, endoplasmic reticulum, or lysosome. The mistargeting of CLDN16 must contribute to loss of Mg^2+^ homeostasis. Patients with FHHNC are most commonly treated with magnesium supplementation, but the effect is incomplete and the serum Mg^2+^ levels remain often low^17^. Finally, the patients develop chronic renal failure and need to undergo renal transplantation. Therefore, there is a need to clarify the pathological mechanisms and develop therapies for FHHNC.

Primaquine, an antimalarial agent, has been reported to interfere with membrane recycling from endosomes to the plasma membrane without neutralizing endosomal pH^18^. Primaquine inhibits the kainate-induced increase in the cell surface expression of kainate receptors in neurons^19^, small-conductance Ca^2+^-activated K^+^ channels in human atrial myocytes^20^, and focal adhesions in vascular smooth muscle cells^21^. In contrast, primaquine inhibits the cellular entry of Clostridium botulinum C2 toxin and Bacillus anthracis lethal toxin^22^. The function of primaquine may be more complicated than has been appreciated previously, but we hypothesized that primaquine may recover the localization and function of CLDN16 mutants found in FHHNC patients.

So far, it has been reported that D97S mutant showed weak expression and only a cytoplasmic localization among all single mutations in the first extracellular loop (ECL) of CLDN16^16^. Therefore, we investigated whether a mutation in D97S affects protein stability, and found that the stability of D97S mutant is lower than that of wild-type (WT). Primaquine increased the protein stability and cell surface localization of D97S mutant. In addition, proteasome activity, lactate dehydrogenase (LDH) release, and cell viability were assessed in D97S mutant-expressing cells because the patients with FHHNC frequently have progressive renal failure. Primaquine decreased the expression of chaperon proteins, proteasome activity, and lactate dehydrogenase release in D97S mutant-expressing cells. Our present results indicated that primaquine may be a new drug in the treatment of a proportion of patients with FHHNC.

## Results

### Effect of D97S mutant CLDN16 expression on endogenous proteins in the cell-cell junctions

**Figure 1:**
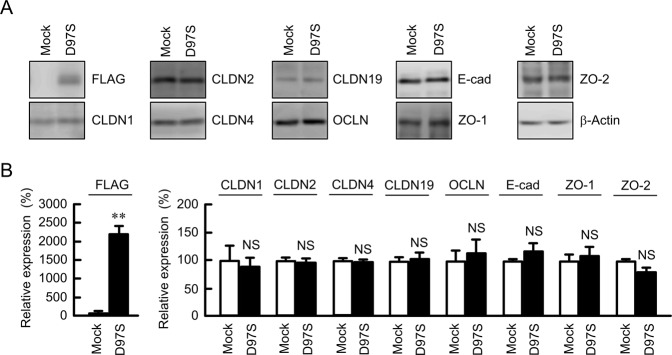
Effect of D97S mutant expression on endogenous junctional protein. (**A**) Stable cell lines of mock or FLAG-tagged D97S mutant CLDN16 in MDCK cells were cultured for 72 h. The cell lysates were blotted with anti-FLAG, anti-CLDN1, anti-CLDN2, anti-CLDN4, anti-occludin (OCLN), anti-E-cadherin (E-cad), anti-ZO-1, anti-ZO-2, and anti-β-actin antibodies. The full-length blot images are shown in Supplementary Fig. S2. (**B**) The band densities of proteins are represented as the percentage of the values in mock cells. n = 4 in four independent experiments. **P < 0.01 compared with mock cells. NS *P* > 0.05.

The D97S mutant CLDN16, which is fused with N-terminal FLAG tag, was stably expressed in Madin-Darby canine kidney (MDCK) cells and confirmed by immunoblotting using an anti-FLAG antibody (Fig. 1A). The expression levels of endogenous junctional proteins including CLDN1, CLDN2, CLDN4, CLDN19, OCLN, E-cadherin, ZO-1, and ZO-2 were not changed by the expression of the D97S mutant. These results were similar to the expression of WT CLDN16^23^.

### Intracellular localization of WT CLDN16 and the D97S mutant in MDCK cells

**Figure 2:**
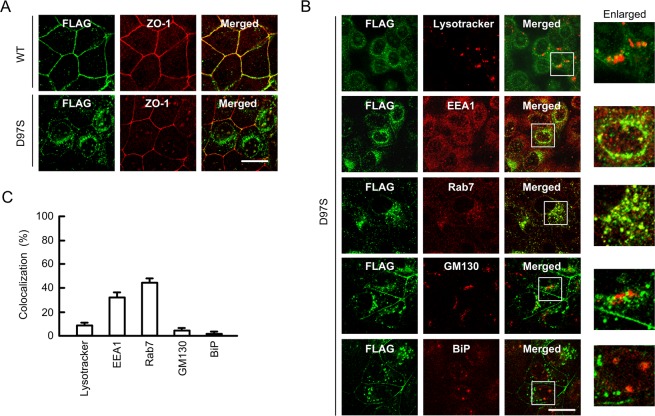
Subcellular localization of WT CLDN16 and the D97S mutant. (**A**) FLAG-tagged WT CLDN16 or the D97S mutant in MDCK cells were incubated with anti-FLAG (CLDN16) and anti-ZO-1 antibodies, followed by incubation with secondary antibodies. Merged images are shown on the right. (**B**) MDCK cells expressing the FLAG-tagged D97S mutant were incubated with anti-FLAG (CLDN16), anti-EEA1, anti-Rab7, anti-GM130, and anti-BiP antibodies or lysotracker. Enlarged images are shown on the right. The scale bar indicates 10 μm. (**B**) The colocalization of FLAG-tagged CLDN16 with organelle markers was shown as the percentage.

The intracellular localization of WT CLDN16 and the D97S mutant was examined using an immunofluorescence staining assay. WT CLDN16 was colocalized with ZO-1, a scaffolding protein of the TJs, in the cell-cell border area (Fig. 2A). In contrast, the D97S mutant was mainly localized in the cytosolic compartment. To specify the subcellular localization of the D97S mutant, a colocalization study with organelle markers was performed. The D97S mutant was partially colocalized with EEA1, an early endosome marker, and Rab7, a late endosome marker, whereas it was not colocalized with lysotracker, a lysosome marker, BiP, an endoplasmic reticulum marker, and GM130, a Golgi apparatus marker (Fig. 2B,C). These results indicated that the endocytosis process of the D97S mutant from the TJs to endosomes may be enhanced.

### Effect of the D97S mutation on the protein stability and degradation of CLDN16

**Figure 3:**
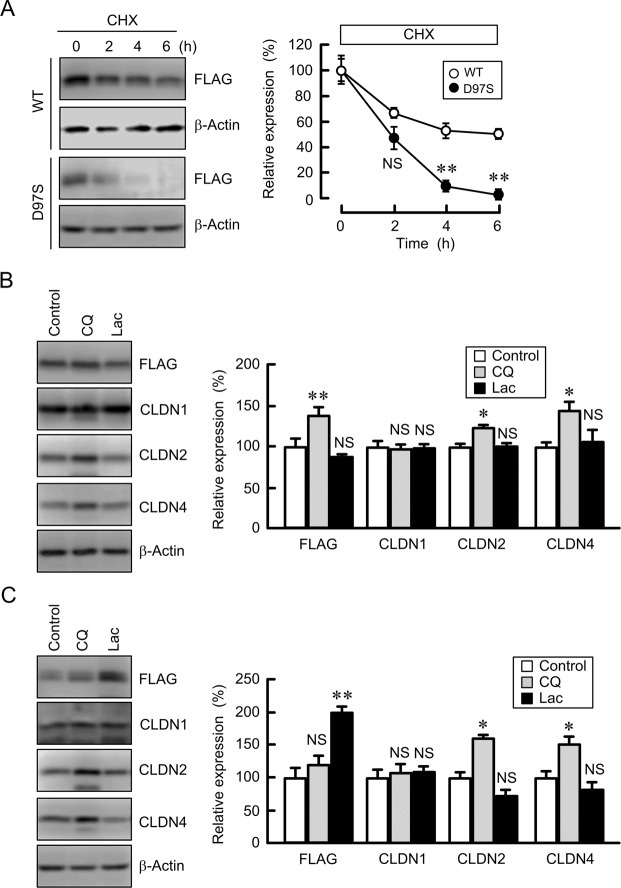
Protein stability of WT CLDN16 and the D97S mutant. MDCK cells stably expressing FLAG-tagged WT CLDN16 or the D97S mutant were treated with cycloheximide (CHX, 5 μM) for the indicated periods. After collecting cell lysates, the aliquots were blotted with anti-FLAG and anti-β-actin antibodies. The band densities of proteins are represented as the percentage of the values at 0 h. (**B**,**C**) MDCK cells stably expressing the FLAG-tagged WT (**B**) or D97S mutant (**C**) were treated with and without chloroquine (CQ, 20 μM) or lactacystin (Lac, 10 μM) for 6 h. The cell lysates were blotted with anti-FLAG, anti-CLDN1, anti-CLDN2, anti-CLDN4, and anti-β-actin antibodies. The band densities of proteins are represented as the percentage of the values in control cells. The full-length blot images are shown in Supplementary Figs S3–S5. n = 4 in four independent experiments. **P < 0.01 and *P < 0.05 compared with control cells. NS *P* > 0.05.

There raises a possibility that a single mutation induces an alteration of protein conformation, resulting in an effect on protein stability. We examined the effect of the D97S mutation on the protein stability of CLDN16. To avoid the effect of protein synthesis, the cells were treated with cycloheximide, an inhibitor of translation. The level of WT CLDN16 was decreased in a time dependent manner (Fig. 3A). The rate of decrease of the D97S mutant was significantly higher than that of WT CLDN16, indicating that the D97S mutation attenuated the protein stability. Similar results were observed in the R131C mutant-expressing cells (Supplementary Fig. S12). Next, we investigated the effect of the D97S mutation on the protein degradation. The majority of intracellular proteins are degraded by the proteasome or lysosomal pathways^24^. The expression level of WT CLDN16 was slightly increased by chloroquine, a lysosome inhibitor, but not by lactacystin, a proteasome inhibitor (Fig. 3B). Similarly, the expression levels of CLDN2 and CLDN4 were increased by chloroquine. The lower molecular bands of CLDN2 and 4 were observed in the chloroquine-treated cells, but there is no report showing that CLDN2 and 4 have splice variants. Therefore, the lower molecular bands were not included in densitometry. In contrast, the expression level of the D97S mutant was increased by lactacystin, but not by chloroquine (Fig. 3C). CLDN1 was changed by neither lactacystin nor chloroquine. These results indicated that the D97S mutant may be mainly degraded in proteasomes, which may be different from the degradation pathway of unmutated proteins of CLDN1, 2, 4, and 16.

### Effects of endocytosis inhibitor and primaquine on the expression and intracellular distribution of the D97S mutant

**Figure 4:**
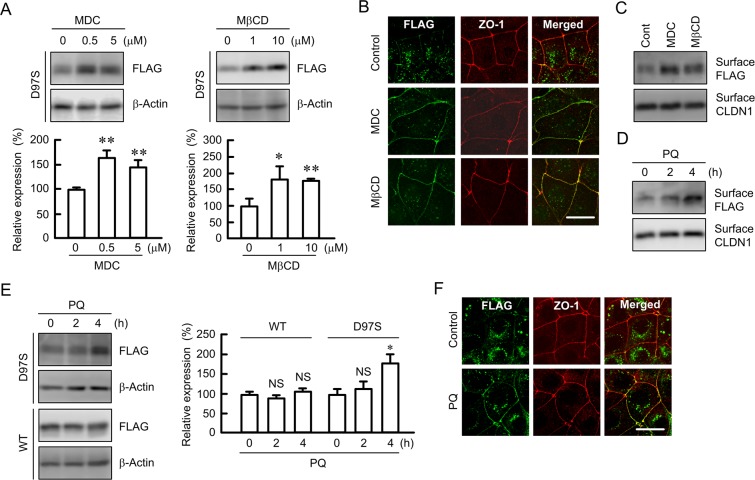
Increase in plasma membrane surface localization of the D97S mutant by endocytosis inhibitor or primaquine treatment. (**A**,**B**) MDCK cells stably expressing the FLAG-tagged D97S mutant were treated with MDC and MβCD for 3 h. After collecting cell lysates, the aliquots were blotted with anti-FLAG and anti-β-actin antibodies. The band densities of proteins are represented relative to the values with 0 μM. (**B**) The cells were stained with anti-FLAG (CLDN16) and anti-ZO-1 antibodies. (**C**,**D**) MDCK cells stably expressing the FLAG-tagged D97S mutant were incubated with primaquine (PQ, 100 μM). The biotinylated proteins were blotted with anti-FLAG and anti-CLDN1 antibodies. (**E**) The cell lysates of MDCK cells expressing the WT or D97S mutant were blotted with anti-FLAG and anti-β-actin antibodies. The band densities of proteins are represented relative to the values at 0 h. The full-length blot images are shown in Supplementary Figs S6–S8. (**F**) The cells were incubated with anti-FLAG (CLDN16) and anti-ZO-1 antibodies. The scale bar indicates 10 μm. n = 4 in four independent experiments. **P < 0.01 and *P < 0.05 compared with 0 μM.

**Figure 5:**
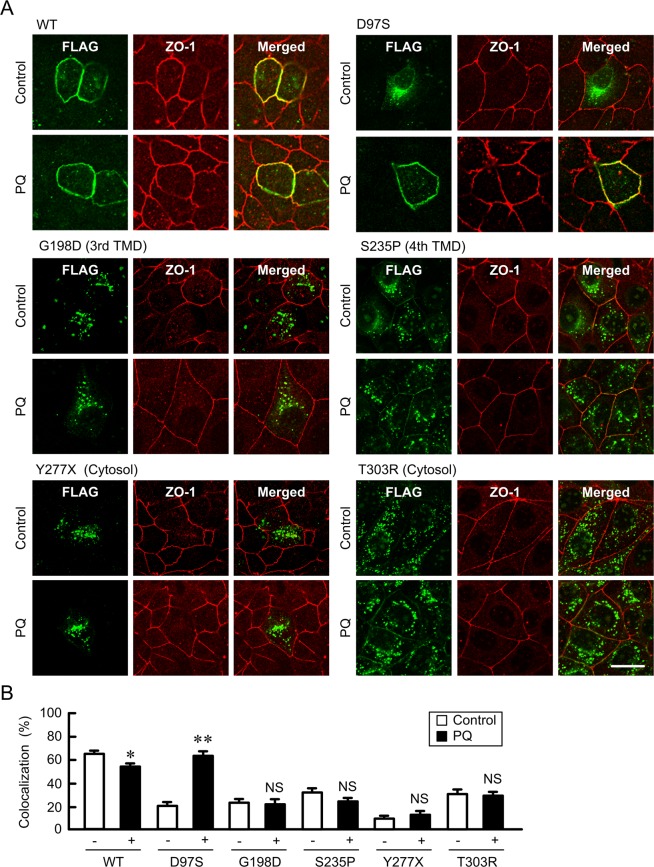
Effect of primaquine on the subcellular localization of CLDN16 mutants. MDCK cells were transiently transfected with FLAG-tagged WT or mutants of CLDN16 including D97S, G198D, S235P, Y277X, and T303R. The cells were incubated for 2 h in the absence or presence of 100 μM primaquine (PQ), and then stained with anti-FLAG (CLDN16) and anti-ZO-1 antibodies. Merged images are shown on the right. The scale bar indicates 10 μm. (**B**) The colocalization of FLAG-tagged CLDN16 with ZO-1 was shown as the percentage. **P < 0.01 and *P < 0.05 compared with control. NS *P* > 0.05.

The expression levels of the D97S mutant were increased by monodansylcadaverine (MDC), a clathrin-mediated endocytosis inhibitor, and methyl-β-cyclodextrin (MβCD), a caveolae-dependent endocytosis inhibitor (Fig. 4A). Immunofluorescence assays showed that the D97S mutant was colocalized with ZO-1 at the TJs in cells treated with MDC and MβCD (Fig. 4B). These results indicated that the D97S mutant is trafficked to the plasma membrane, but it can also be quickly internalized into the cytosolic compartment. To support the elevation of the D97S mutant in the plasma membrane, we performed biotinylation assay, which allows the detection of cell surface protein expression. The cell surface expression of the D97S mutant was increased by primaquine in a time-dependent manner, whereas that of CLDN1 was constant (Fig. 4C). Similarly, primaquine increased the cell surface expression of the D97S mutant (Fig. 4D). The total amount of the D97S mutant was increased by primaquine, but that of WT was not (Fig. 4E). Immunofluorescence assays showed that primaquine increased the tight junctional localization of the D97S mutant (Fig. 4F). Similarly, primaquine increased the tight junctional localization of the D97S mutant in another stable and transient clones (data not shown). To clarify the effect of primaquine on other mutants of CLDN16, we investigated the localization of these mutants using transient expression assays. All mutants were mainly distributed in the cytosolic compartment similar to the D97S mutant under control conditions (Figs 5 and S14A). Primaquine increased the tight junctional localization of the D97S and R131C mutants, which have mutations in transmembrane domain (TMD) 1, whereas it did not change that of the G198D and S235P mutants, which have mutations in TMD3 or TMD4, or that in the Y277X and T303R mutants, which have mutations in the carboxyl cytosolic domain. Primaquine significantly decreased the tight junctional localization of WT, but the effect was weak.

### Effect of primaquine on protein stability and ubiquitination of the D97S mutant

**Figure 6:**
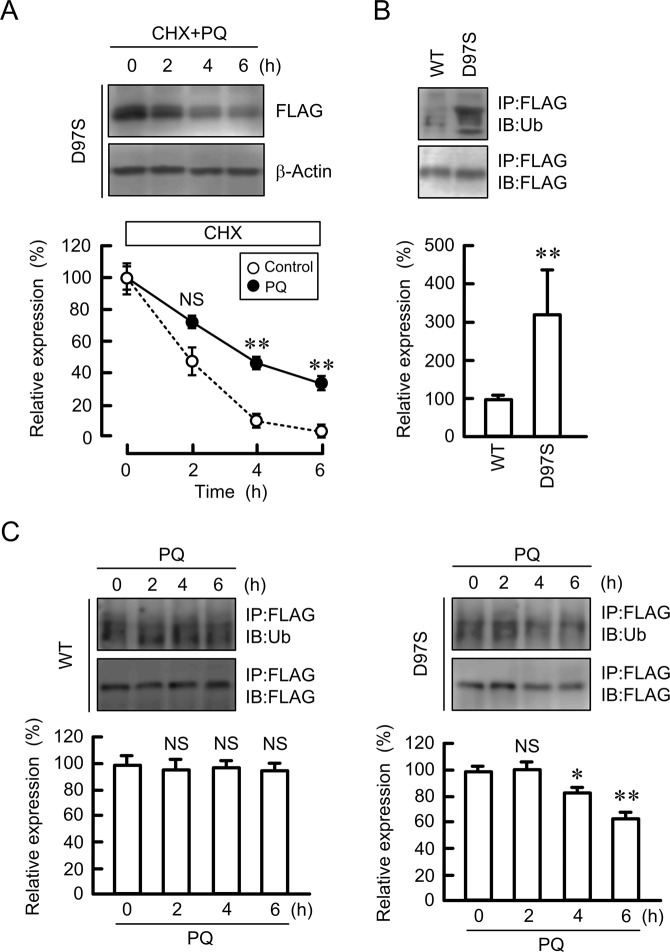
Effect of primaquine on protein stability and ubiquitination of the D97S mutant. (**A**) MDCK cells expressing the FLAG-tagged D97S mutant were treated with cycloheximide (CHX, 5 μM) and primaquine (100 μM) for the indicated periods. After collecting cell lysates, the aliquots were blotted with anti-FLAG and anti-β-actin antibodies. The band densities of proteins are represented as the percentage of the values at 0 h. The dashed line shows the relative expression of D97S mutant in the absence of primaquine as indicated in Fig. 3A. (**B**) The cell lysates isolated from MDCK cells expressing FLAG-tagged WT CLDN16 and the D97S mutant were immunoprecipitated with protein G sepharose beads and anti-FLAG antibody. Immune pellets were blotted with anti-ubiquitin (Ub) and anti-FLAG antibodies. The band densities of ubiquitinated CLDN16 are represented as the percentage of the values in WT. (**C**) MDCK cells expressing the FLAG-tagged WT CLDN16 or D97S mutant were incubated with 100 μM primaquine (PQ) for the indicated periods. The cell lysates were immunoprecipitated with anti-FLAG antibody. Immune pellets were blotted with anti-Ub and anti-FLAG antibodies. The band densities of ubiquitinated CLDN16 are represented as the percentage of the values at 0 h. The full-length blot images are shown in Supplementary Figs S9 and S10. n = 4 in four independent experiments. **P < 0.01 compared with 0 h or WT. NS *P* > 0.05 compared with control or 0 h.

The rate of decrease in the D97S mutant was significantly attenuated by primaquine (Fig. 6A). As described in Fig. 4F, primaquine increased the tight junctional localization of the D97S mutant. These results indicated that primaquine may enhance stabilization of the D97S mutant in the TJs. The endocytosis of WT CLDN16 from the TJs to cytosolic compartment is regulated by a ubiquitination process^25^. The ubiquitination level of the D97S mutant was higher than that of WT CLDN16 (Fig. 6B). Primaquine significantly decreased the ubiquitination level of the D97S mutant, whereas it did not change that of WT CLDN16 (Fig. 6C). These results indicated that de-ubiquitination may be involved in the stabilization of the D97S mutant by primaquine.

### Effect of primaquine on the function of the D97S mutant

**Figure 7:**
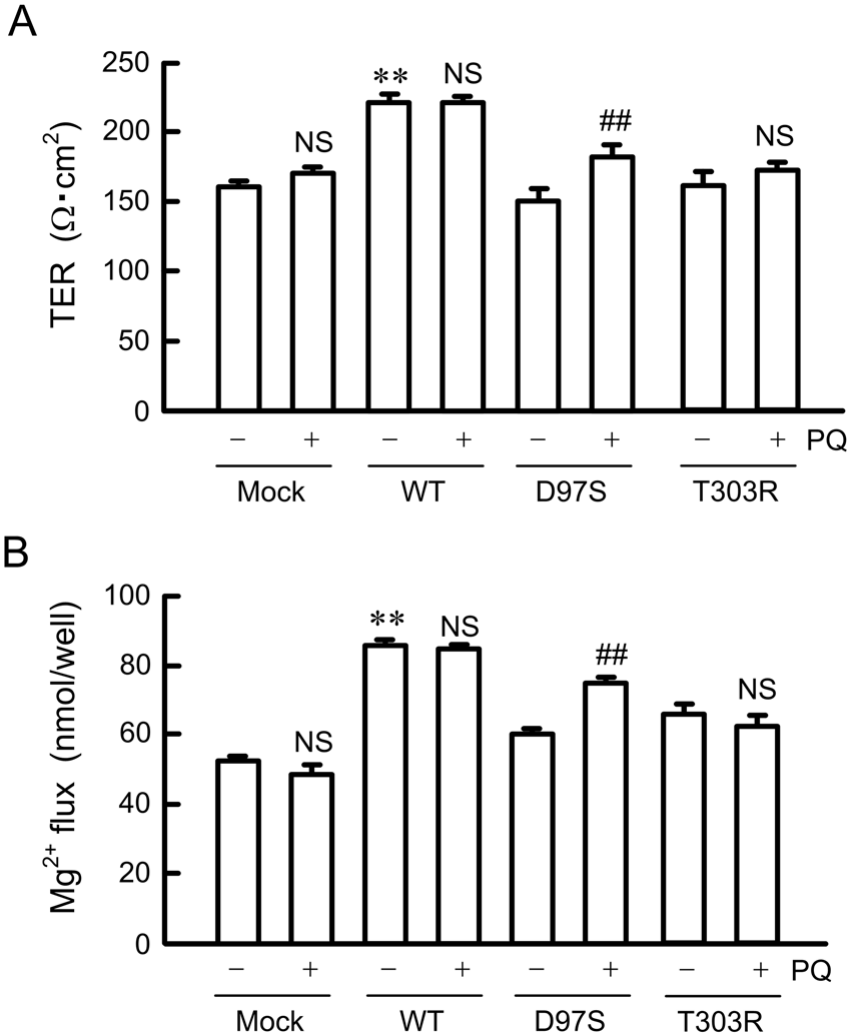
Effects of primaquine on paracellular permeability. FLAG-tagged WT CLDN16, D97S, or T303R mutant were transiently transfected in MDCK cells and cultured on transwell inserts for 96 h, then incubated for 2 h in the absence or presence of primaquine (PQ, 100 μM). (**A**,**C**) TER values were measured using a Millicell-ERS epithelial volt-ohmmeter and expressed as resistance (Ω·cm^2^). (**B**,**D**) The transepithelial permeability to Mg^2+^ for 1 h was measured using XB-1. n = 4 in four independent experiments. **P < 0.01, *P < 0.05, and NS *P* > 0.05 compared without PQ.

The tight junctional localization of WT CLDN16 increases TER and transepithelial Mg^2+^ flux^26^. TER and transepithelial Mg^2+^ flux in cells-expressing WT CLDN16 was not changed by primaquine (Fig. 7). In contrast, primaquine significantly increased TER and transepithelial Mg^2+^ flux in D97S mutant-expressing cells. Similar results were observed in the R131C mutant-expressing cells (Supplementary Fig. S14B and S14C), but not in the T303R mutant-expressing cells. These results indicated that primaquine may rescue the function of the D97S and R131C mutants.

### Effect of primaquine on protein aggregation of the D97S mutant

**Figure 8:**
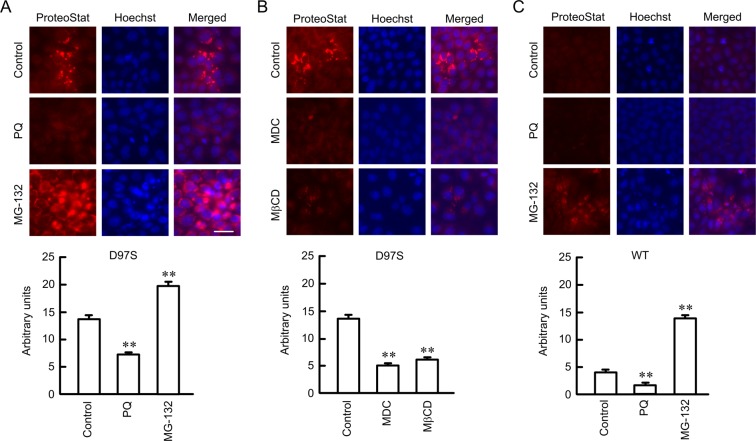
Decrease in aggresome formation by primaquine and endocytosis inhibitors. (**A**,**B**) The FLAG-tagged D97S mutant-expressing cells were treated with primaquine (100 μM), MDC (5 μM), MβCD (10 μM) and MG-132 (10 μM). (**C**) The FLAG-tagged WT CLDN16-expressing cells were incubated with 100 μM primaquine and MG-132 (10 μM) for 6 h. Then, the cells were incubated with ProteoStat Aggresome Detection Reagent and Hoechst 33342 for 1 h. The fluorescence images were collected using a confocal microscope. The fluorescence intensity of aggresome signal (red) is shown as arbitrary units. n = 4 in four independent experiments. The scale bar indicates 10 μm. **P < 0.01 compared with control.

Overexpression of misfolded proteins leads to the formation of aggresome^27^. Aggresome formation was examined using a ProteoStat Aggresome Detection kit. Primaquine decreased the red fluorescent signal of aggresome formation in D97S mutant-expressing cells (Fig. 8). Similarly, MDC and MβCD decreased aggresome formation. These results were inversely proportional to tight junctional localization of the D97S mutant (Fig. 4B,F). In contrast, MG-132, a proteasome inhibitor, significantly increased aggresome formation. Aggresome formation in WT CLDN16-expressing cells was lower than that in the D97S mutant, whereas it was increased by MG-132. Immunofluorescence assays showed that aggresome is colocalized with Rab7 (Supplementary Fig. 1). These results indicated that the D97S mutant may enhance the formation of aggresome and the misfolded proteins are degraded by proteasome.

### Effect of primaquine on chaperon expression, proteasome activity, and cell injury

**Figure 9:**
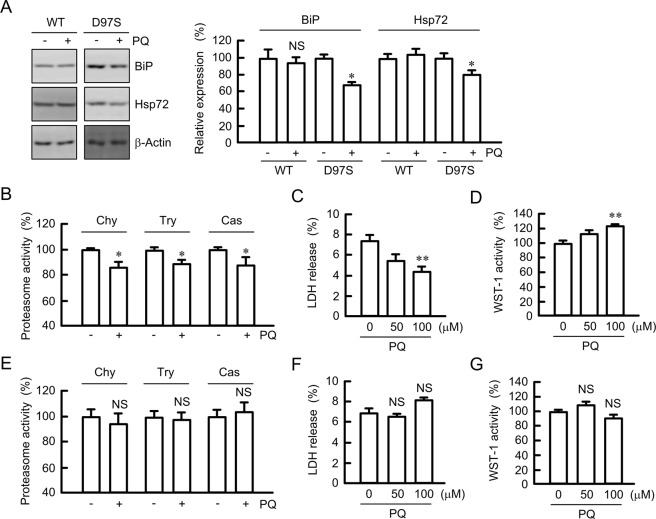
Decrease in chaperone protein, proteasome activity, and cell injury by primaquine. (**A**) The FLAG-tagged WT CLDN16- or D97S mutant-expressing cells were treated with primaquine (PQ, 100 μM) for 6 h. After collecting cell lysates, the aliquots were blotted with anti-BiP, anti-Hsp72, and anti-β-actin antibodies. The band densities of proteins are represented relative to the values without PQ. The full-length blot images are shown in Supplementary Fig. S11. (**B**,**E**) The cytosolic fraction was isolated from D97S mutant-expressing cells (**B**) or WT CLDN16-expressing (**E**) using a passive buffer. Chymotrypsin-like (Chy), trypsin-like (Try), and caspase-like (Cas) proteasome activities were measured using each selective substrates. (**C**,**D**,**F** and **G**) MDCK cells expressing the FLAG-tagged D97S mutant (**C**,**D**) or WT CLDN16 (**F**,**G**) were incubated with 100 μM PQ for 24 h. Cell injury was estimated by LDH release into the media and WST-1 activity. n = 4 in four independent experiments. **P < 0.01, *P < 0.05, and NS *P* > 0.05 compared with 0 h or without PQ.

The folding of proteins is regulated by chaperons including BiP and the Hsp70 family^28^. The expression levels of BiP and Hsp72 were decreased by primaquine in D97S mutant-expressing cells, but not in WT CLDN16-expressing cells (Fig. 9A). The activity of proteasomes is often increased by elevated levels of misfolded proteins and their aggregates^29^. Primaquine decreased chymotrypsin-like, trypsin-like, and caspase-like proteasome activities in D97S mutant-expressing cells (Fig. 9B). Similar results were observed in the R131C mutant-expressing cells (Supplementary Fig. S15). Some mutant proteins produce misfolded proteins and their aggregates, resulting in the progression of cell injury^30^. Primaquine decreased LDH release, whereas it increased WST-1 activity in D97S mutant-expressing cells (Fig. 9C,D), indicating the primaquine decreased the cell injury. In contrast, primaquine did not significantly change proteasome activity, LDH release, and WST-1 activity in WT CLDN16-expressing cells (Fig. 9E–G). These results indicated that the reduction in aggregated D97S mutant by primaquine may attenuate proteasome activity and cell injury.

## Discussion

Dysfunction of CLDN16 and CLDN19 is involved in the pathogenesis of genetic hypomagnesemia. Over 40 and 10 different mutations have been currently identified in the *CLDN16* and *CLDN19* genes, respectively^13,16,31,32^. Mutations in the *CLDN16* gene mainly occur in the ECL and TMD. Although there is a difference in the degree, the patients with mutation in *CLDN16* gene show hypomagnesemia independently of the mutation sites^33^. The function and intracellular localization are different in each mutant. The mutants of CDLN16 distributed in the TJs have full or partial function after being transfected into LLC-PK_1_, a porcine proximal tubular cell line^16^, and MDCK-C7 cells^13^. In contrast, the mutants that mislocalized to intracellular compartments including the endoplasmic reticulum, Golgi apparatus, and lysosome, lose their function. Our results showed that the D97S, R131C, G198D, S235P, Y277X, and T303R mutants of CLDN16 were mainly localized in the cytosolic compartment (Figs 2 and 5). The D97S mutant was mainly localized the endosome in MDCK cells (Fig. 2B), whereas the mutant was localized in the endoplasmic reticulum (ER) in LLC-PK_1_ cells^16^. In addition, the G198D mutant was expressed in the cytosolic compartment in MDCK cells, whereas it was not detected in LLC-PK_1_ cells. At present, we do not know the reason for the difference, but cell type may affect on expression and subcellular localization of mutants.

Several genetic diseases induce the mislocalization of membrane proteins. The mislocalization of misfolded mutant ΔF508-cystic fibrosis transmembrane conductance regulator was restored by chemical chaperones such as sodium 4-phenylbutyrate^34^ and quinazoline derivate^35^. The maturation, cell-surface expression, and function of a vasopressin V3 receptor mutant were rescued by SSR149415^36^. In the present study, we found that mislocalization of the D97S mutant of CLDN16 was restored by primaquine. It has been reported that the recycling of transmembrane CD4 receptor is sensitive to primaquine in transfected Chinese hamster ovary cells^21^. Primaquine increased the protein stability and cell surface localization of the D97S mutant (Figs 4D,E, 5, and 6A). Therefore, we suggested that the effect of primaquine was mainly caused by the inhibition of endocytosis of the D97S mutant. The mislocalization and function of R131C were also recovered by primaquine, but other mutants were not. There is a possibility that primaquine recover the localization and function of first ECL of CLDN16 mutants. However, biochemical properties of other mutants including R131C and those in other renal cells have not been clarified. We need further study to clarify the effect of primaquine on all mutants in detail using various renal tubular epithelial cells.

CLDN16 can interact with CLDN19, which are colocalized in the TJs^37^. Our data indicate that the signal of CLDN19 was weak, but it may be endogenously expressed in MDCK cells (Fig. 1). Hou *et al*. reported that the knockdown of CLDN19 by siRNA in mice causes a loss of tight junctional CLDN16 in the TAL^37^, whereas CLDN16 is localized at TJs in MDCK and LLC-PK1 cells without expressing CLDN19. We do not know whether CLDN19 is necessary for the tight junctional localization of CLDN16 in MDCK cells. The single mutation in amino acids located in the first ECL suggests the inhibition of homo-oligomerization of CLDN16 or hetero-oligomerization with CLDN19, leading to the elevation of instability of the mutants in the TJs. Primaquine increased transepithelial permeability to Mg^2+^ in the D97S or R131C mutant-expressing cells (Fig. 7). The cation permeable pore of CLDN16 is formed by the negatively charged amino acids located in the first ECL, which are different from D97 and R131. We do not know whether primaquine induces the formation of homo-oligomerization of CLDN16 or hetero-oligomerization with CLDN19, but the D97S mutant must form a Mg^2+^-permeable pore in the TJs. In addition, TER was increased by WT CLDN16 expression and the D97S mutant treated with primaquine. Therefore, it cannot be denied that CLDN16 and primaquine may up-regulate the barrier forming CLDNs or down-regulate channel forming CLDNs. Further studies are needed to clarify how CLDN16 increases TER and transepithelial permeability to Mg^2+^, but we found for the first time that primaquine rescues the function of the D97S mutant.

So far, we reported that the tight junctional localization of WT CLDN16 is suppressed by treatment with 200 μM primaquine^26^. In contrast, we found that the tight junctional localization of the D97S mutant were rescued by 100 μM primaquine in the present study. These results showed that primaquine has opposite effects depending on the concentration or target molecules. Primaquine may not directly increase exocytosis or inhibit endocytosis of the CLDN16 mutants. Because the ubiquitination level of the D97S mutant was higher than that of WT CLDN16, which was decreased by primaquine. We suggested that primaquine inhibits the association between CLDN16 and the regulatory factor of endocytosis.

FHHNC is characterized by not only by progressive renal failure of renal reabsorption of Mg^2+^ and Ca^2+^, but also by abnormal reabsorption of divalent cations, and nephrocalcinosis and renal stone formation, leading to chronic renal failure. Hypercalciuria may be one of the causes of renal failure in FHHNC patients, but the exact mechanism of the development of renal failure have not been clarified fully. Hypomagnesemia is observed in the CLDN16 RNAi mice^38^ and CLDN16 targeted deletion mice^39^, but renal failure is not. This means that the losses of function and expression of CLDN16 may not be a cause of renal failure. The accumulation of misfolded or damaged proteins causes cell damage^40^. Increased proteasome activity could be an adaptive response to the augmented accumulation of misfolded proteins. The red fluorescence of ProteoStat was colocalized with Rab7 (Supplementary Fig. 1). Bocock *et al*.^41^ reported that proteasome can degrade the proteins in the endosomal membranes. We suggest that the aggregated D97S mutant is present in the late endosome, followed by transported to proteasome. The aggregated proteins were decreased by primaquine in D97S mutant-expressing cells (Fig. 8A). In addition, LDH release and WST-1 assays suggest that primaquine reduces the cellular injury in D97S mutant-expressing cells (Fig. 9). When the accumulation of aggregated proteins exceeds the threshold of degradation by proteasomes may induce renal tubular damage. The expression levels of unmutated proteins of CLDN2, 4, and 16 were increased by chloroquine, indicating that these proteins were degraded in the lysosome. The degradation mechanism of the D97S mutant may be different from that of WT CLDN16. The lower molecular bands of CLDN2 and CLDN4 were observed in the chloroquine-treated cells. There is no report showing that CLDN2 and CLDN4 have splicing variants, and these molecules may be usually degraded in the lysosome under control conditions.

**Figure 10:**
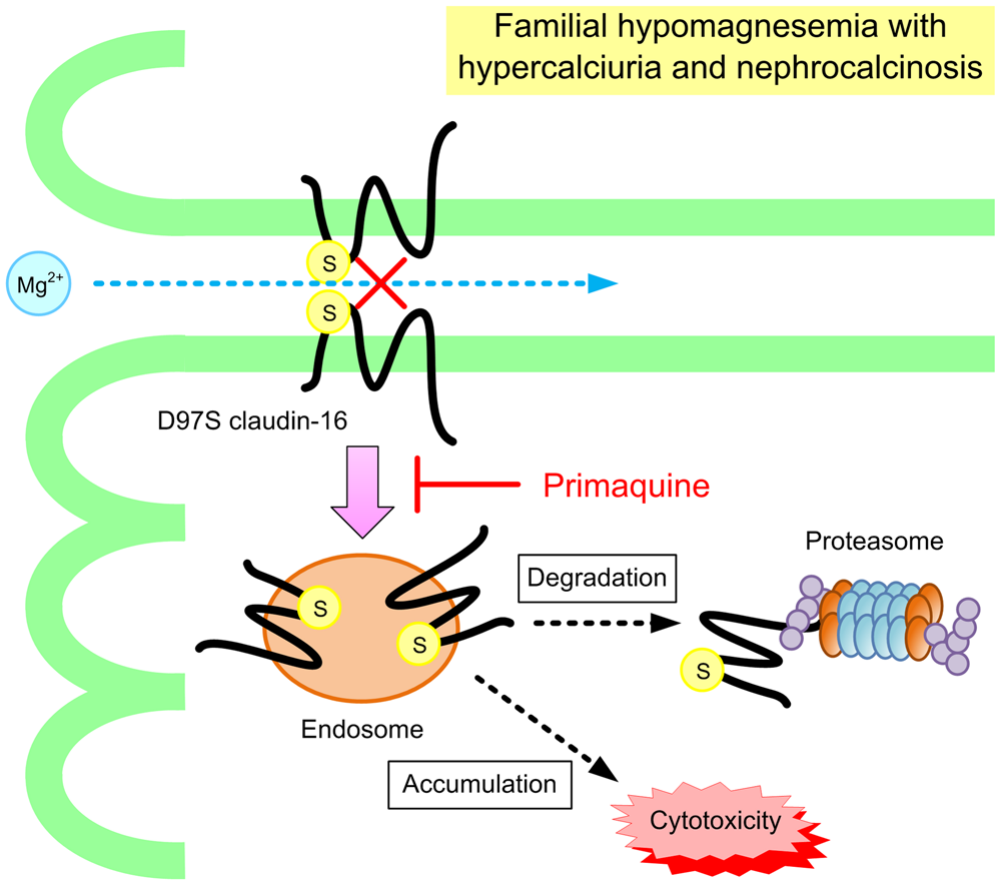
A schematic model for the internalization of D97S mutant CLDN16.

Taken together, the results of the present study demonstrated that the D97S mutant has lower protein stability compared with WT CLDN16. The mutant was mainly distributed in the endosome and degraded by proteasomes. Primaquine increased the tight junctional localization of the D97S mutant, leading to the elevation of TER and transepithelial Mg^2+^ flux. In addition, primaquine decreased the formation of aggresome and cell injury. Our data are summarized in Fig. 10. We suggest that primaquine may be the basis for an effective treatment drug in patients with mutations in the first ECL of CLDN16.

## Materials and Methods

### Materials

Rabbit anti-CLDN1 (71–7800), anti-CLDN2 (51–6100), and anti-ZO-1 (61–7300), and mouse anti-CLDN4 (32–9400) antibodies were obtained from Zymed Laboratories (South San Francisco, CA, USA). Mouse and rabbit anti-FLAG (018-22381 and PM020) antibodies were from Wako Pure Chemical Industries (Osaka, Japan) and Medical & Biological Laboratories Co. (Nagoya, Japan), respectively. Rabbit anti-Rab7 (9367) antibody was from Cell Signaling Technology (Beverly, MA, USA). Goat anti-β-actin (sc-1615), anti-occludin (sc-8145), and anti-ZO-2 (sc-8148) antibodies were from Santa Cruz Biotechnology (Dallas, TX, USA). Rabbit anti-EEA1 (PA1-063) and anti-CLDN19 (SAB2100440) antibodies were from Affinity BioReagents (Golden, CO, USA) and Sigma-Aldrich (St. Louis, MO, USA), respectively. Mouse anti-BiP (610978), anti-E-cadherin (610181), and anti-GM130 (610822) antibodies were from BD Biosciences Clontech (San Jose, CA, USA). Mouse anti-ubiquitin (PW0150) antibody was from Biomol GmbH (Waidmannstr, Germany). LysoTracker Red DND-99 was from Thermo Fisher Scientific (Waltham, MA, USA). Lipofectamine 2000 was from Invitrogen (Carlsbad, CA, USA). All other reagents were of the highest grade of purity available.

### Plasmid DNA construction

FLAG-tagged CLDN16/pTRE2hyg vector was generated as described previously^25^. An N-terminal FLAG tag is added to CLDN16. The mutants of CLDN16 (D97S, R131C, G198D, S235P, Y277X, and T303R) were generated using a KOD-Plus-mutagenesis kit (Toyobo Life Science, Osaka, Japan). Primer pairs are listed in Table 1.

**Table 1:** Primer pairs for point mutation of CLDN16.

Name	Direction	Sequence
D97S	Forward	5′-AGCTGTTGGATGGTGAATGCTGATGACT-3′
D97S	Reverse	5′-AGTCCAGGTGGCCACAATCAAAAAC-3′
R131C	Forward	5′-TGCACCTGTGATGAGTACGATTCCATAC-3′
R131C	Reverse	5′-AATCCCATCAAAAGCATTTGTGACG-3′
G198D	Forward	5′-GATACCCCAGGAATCATTGGCTCTGTGT-3′
G198D	Reverse	5′-TGCTATTAGTAACGTGGCTCCAGCA-3′
S235P	Forward	5′-CCCTGTTGGCTCGGAATGGCTGGGTCTC-3′
S235P	Reverse	5′-CCAACCAAATTTATATTGGATACCA-3′
Y277X	Forward	5′-TAATCAGCCGCGGGTGTTTCCATGGCCA-3′
Y277X	Reverse	5′-GGCTTTCCTCAAGGAATAAGGATAG-3′
T303R	Forward	5′-AGAAGGGTGTAAGTCGACGATATCTCTA-3′
T303R	Reverse	5′-GTCTACAGCATACATTTTGGCCGTC-3′

### Cell culture and transfection

The type II MDCK cell line (MDCK Tet-OFF, BD Biosciences Clontech) were grown in Dulbecco’s modified Eagle’s medium (Sigma-Aldrich, St. Louis, MO, USA) supplemented with 5% fetal bovine serum, 0.07 mg/ml penicillin-G potassium, 0.14 mg/ml streptomycin sulfate, 0.1 mg/ml G418, and 0.1 mg/ml hygromycin B in a 5% CO_2_ atmosphere at 37 °C. Plasmid DNA was transfected into cells using Lipofectamine 2000 as recommended by the manufacturer. Stable transfectants of empty vector (mock), WT CLDN16, and the D97S mutant were maintained in the continuous presence of the selecting drug.

### Preparation of cell lysates and immunoprecipitation

Confluent MDCK cells were scraped into cold phosphate-buffered saline (PBS) and collected by centrifugation. The cells were lysed in a RIPA buffer containing 150 mM NaCl, 0.5 mM EDTA, 1% Triton X-100, 50 mM Tris-HCl (pH 8.0), and protease inhibitor cocktail (Sigma-Aldrich), and were then sonicated for 20 s. The nuclear fraction was removed by centrifugation at 1,000 × *g* for 5 min, the supernatant was used as cell lysates. Immunoprecipitation assay was performed using cell lysates, protein G sepharose beads, and anti-FLAG antibody. By centrifugation at 6,000 × *g* for 1 min, the immune pellets were washed four times with RIPA buffer. In a biotinylation assay, plasma membrane surface proteins were biotinylated as described previously^42^. The cell lysates, immunoprecipitants, and biotinylated proteins were diluted in sample buffer for sodium dodecyl sulfate (SDS)-polyacrylamide gel electrophoresis (PAGE). The protein concentrations were measured by Bradford assay in which bovine serum albumin was used as a standard.

### SDS-PAGE and immunoblotting

SDS-PAGE was performed as described previously^43^. Briefly, samples (30 μg/lane) were applied to the SDS-polyacrylamide gel. After blotting proteins onto a polyvinylidene difluoride (PVDF) membrane and incubated with each primary antibody (1:1000 dilution) followed by a peroxidase-conjugated secondary antibody (1:5000 dilution). Finally, the blots were incubated in EzWestLumi plus (ATTO Corporation, Tokyo, Japan) and scanned using a C-DiGit Blot Scanner (LI-COR Biotechnology, Lincoln, NE). Band density was quantified using ImageJ software (National Institute of Health software). β-Actin was used for normalization.

### Measurement of paracellular permeability

MDCK cells expressing FLAG-tagged CLDN16 were cultured at confluent densities on transwell inserts (Corning Incorporated-Life Sciences, Acton, MA, USA). TER values was measured using a Millicell-ERS epithelial volt-ohmmeter (Millipore, Billerica, MA, USA) and calculated as described previously^23^. The transepithelial permeability to Mg^2+^ from the apical to basal compartments was quantified by a colorimetric method using Xylidyl blue-I (XB-I). The apical and basal compartments were filled with a transport buffer containing 140 mM NaCl, 5.8 mM KCl, 0.34 mM Na_2_HPO_4_, 0.44 mM KH_2_PO_4_, 1 mM CaCl_2_, 25 mM glucose, and 20 mM Hepes (pH 7.4). At time 0, 10 mM MgCl_2_ was added into the apical compartment. After incubation at 4 °C for 1 h, the buffer in the basal compartment was collected. The absorbance of XB-I at 520 nm was measured under alkaline conditions using a Bio-Spec Mini spectrophotometer (Shimadzu, Tokyo, Japan). Mg^2+^ concentration was calculated using a calibration curve.

### Confocal microscopy

MDCK cells expressing FLAG-tagged CLDN16 were stained as described previously^44^. The primary and secondary antibodies are diluted 1:100. The fluorescence images were collected on an LSM 700 confocal microscope system (Carl Zeiss, Germany) with an appropriate filter set for Dylight 488, Dylight 550, and LysoTracker Red DND-99. The merged area between FLAG-tagged CLDN16 and organelle markers or ZO-1 was shown as percentage of total intensities of organelle markers or ZO-1, respectively.

### Assessment of aggresome formation

Protein aggregation was measured using a ProteoStat Aggresome Detection kit accordance with the manufacturer’s instructions (Enzo Life Sciences, Farmingdale, NY, USA). The fluorescence images were captured using a fluorescence microscope (BZ-9000, Keyence, Osaka, Japan). The fluorescence intensity of ProteoStat dye was calculated using ImageJ software.

### Measurement of proteasome activity

Proteasome activity was examined using fluorogenic peptides N-Suc-LLVY-AMC, Z-LRR-AMC, and Z-LLE-AMC as substrates to measure chymotrypsin-like, trypsin-like, and caspase-like peptidase activities, respectively. Briefly, the cells were washed twice with PBS and homogenized in a passive buffer (Promega Corporation, Madison, WI, USA). After sonication for 20 s, the supernatant was collected by centrifugation at 16,000 × g for 10 min. The aliquots were diluted in an assay buffer containing 250 mM sucrose, 10 mM NaCl, 1.5 mM MgCl_2_, 1 mM EDTA, 1 mM EGTA, 2 mM ATP, 5 mM dithiothreitol, 50 mM Hepes (pH 7.8), and 10 μM proteasome substrates, and then the reaction mixture was incubated for 1 h at 37 °C. The proteolytic activity was measured by monitoring the formation of the fluorescent AMC (excitation wavelength 380 nm and emission wavelength 460 nm).

### Cell injury assay

Cytotoxicity was assessed by LDH release and 2-(4-iodophenyl)-3-(4-nitrophenyl)-5-(2,4-disulfophenyl)-2*H*-tetrazolium, monosodium salt (WST-1) activity. Cells were seeded at 4 × 10^3^ cells/well in a 96-well plate. The cells were incubated with primaquine for 24 h and then LDH release was measured using an LDH Cytotoxicity Detection kit (Wako Pure Chemical Industries). Cell viability was assessed using a WST-1 Cell Counting kit (Dojindo Laboratories, Kumamoto, Japan).

### Statistics

Results are presented as the mean ± S.E. Differences between groups were analyzed using one-way ANOVA. Corrections for multiple comparisons were made using Tukey’s multiple comparison test. Comparisons between two groups were made using Student’s *t* test. Significance was set at a *p* value < 0.05.

## Supplementary information

Supplementary Dataset 1
