# Supplementary material for: Rescue of tight junctional localization of a claudin-16 mutant D97S by antimalarial medicine primaquine in Madin-Darby canine kidney cells
Source: Sci Rep. 2019 Jul 4;9:9647. doi: 10.1038/s41598-019-46250-4 (PMC6609605; doi:10.1038/s41598-019-46250-4)
Supplement: Supplementary file 1 — Supplementary Dataset 1 [file 41598_2019_46250_MOESM1_ESM.pdf]

Supplementary Information

Rescue of tight junctional localization of a claudin-16 mutant D97S by antimalarial medicine primaquine in Madin-Darby canine kidney cells

Kana Marunaka[1], Naoko Fujii[1], Toru Kimura[2], Takumi Furuta[3], Hajime Hasegawa[4], Toshiyuki Matsunaga[1], Satoshi Endo[1] & Akira Ikari[1,*]

[1] Laboratory of Biochemistry, Department of Biopharmaceutical Sciences, Gifu Pharmaceutical University, Gifu, Japan

[2]Department of Pharmacology and Toxicology, Kyorin University School of Medicine, Tokyo, Japan

[3]Department of Pharmaceutical Chemistry, Kyoto Pharmaceutical University, Kyoto, Japan

[4]Saitama Medical Center, Saitama Medical University, Saitama, Japan

Running title: Primaquine restores localization of claudin-16 mutant D97S

*Correspondence and requests for materials should be addressed to A. I.

(email: ikari@gifu-pu.ac.jp)

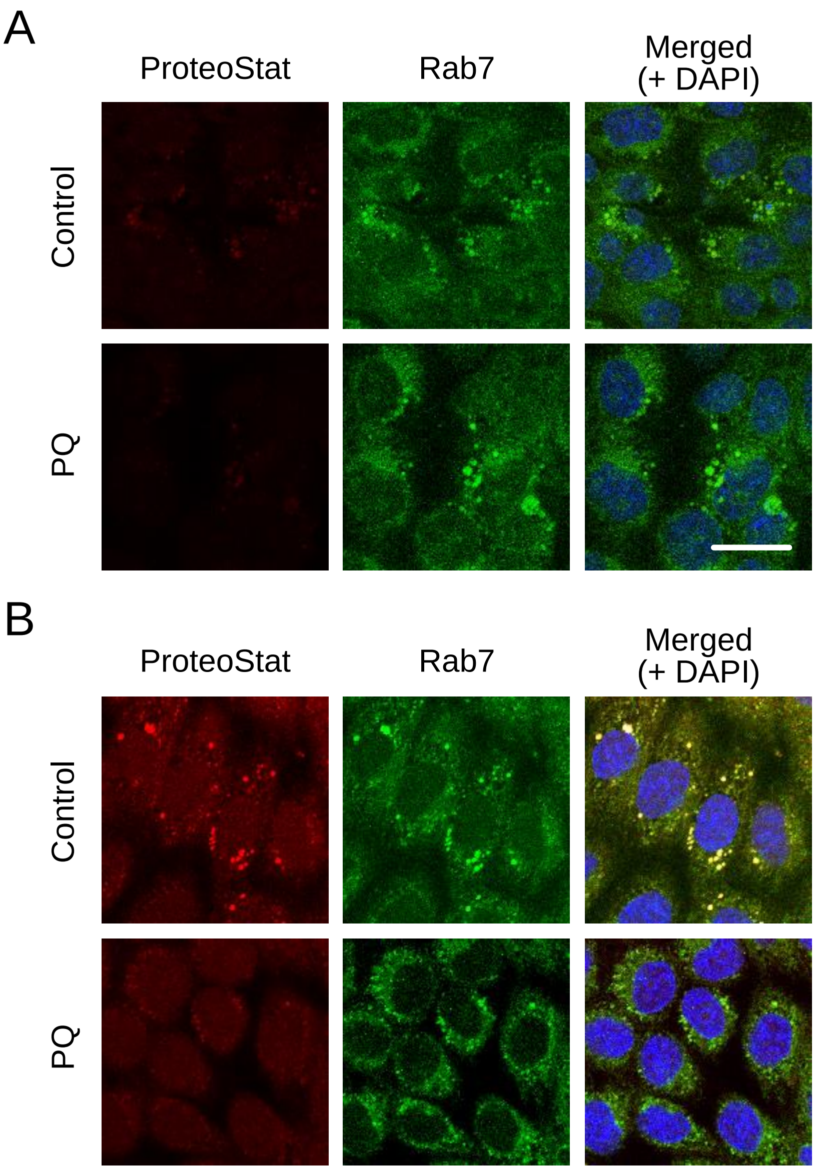

Supplementary figure S1. Localization of aggresome in the lysosome. MDCK cells expressing FLAG-tagged WT CLDN16 (A) or the D97S mutant (B) were incubated for 4 h in the absence and presence of 100 μM primaquine (PQ), and then stained with ProteoStat (red) and anti-Rab7 antibodies (green). Merged images with DAPI are shown on the right. The scale bar represents 10 μm.

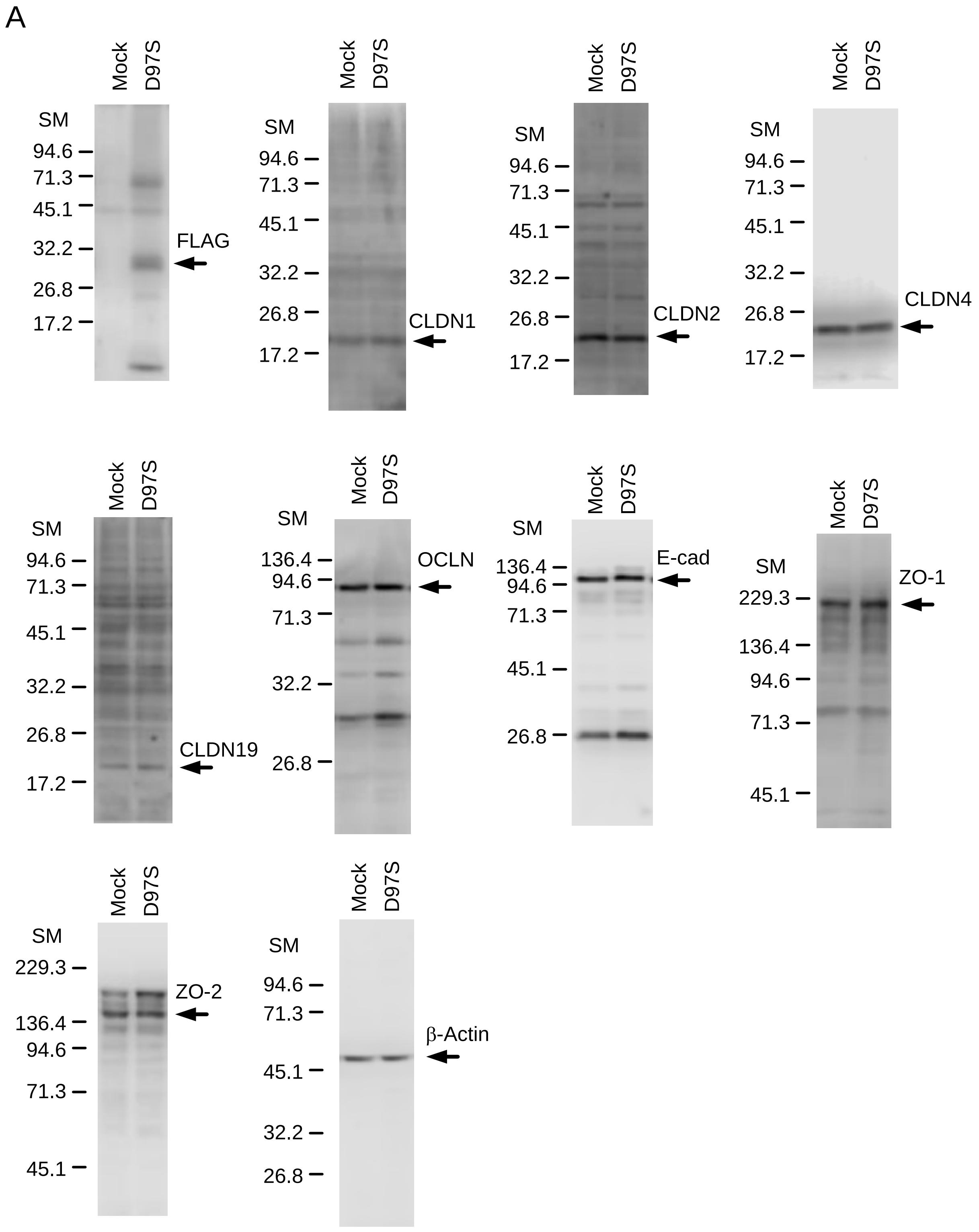

Supplementary Figure S2. Full images of the blots from figure 1A.

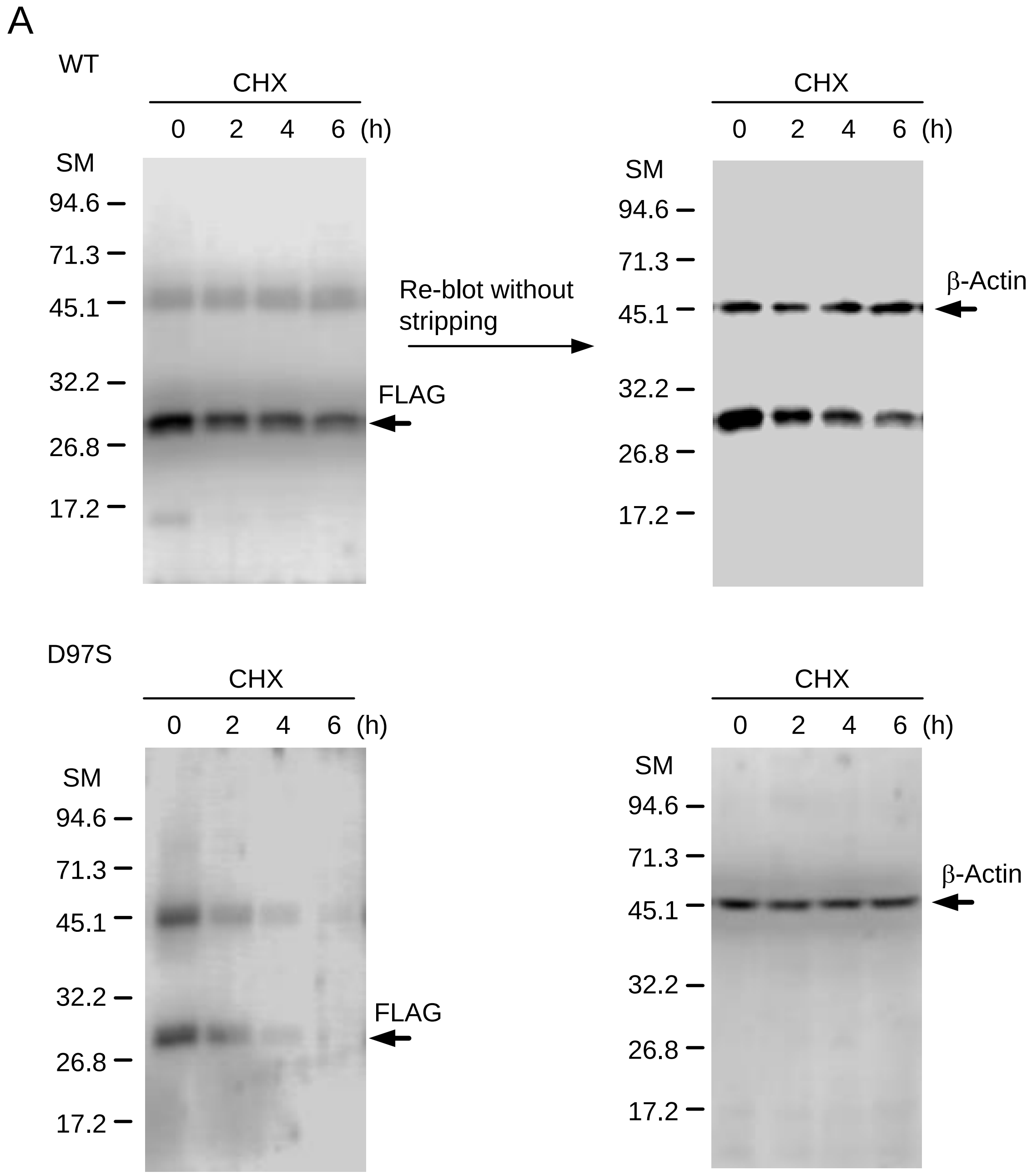

Supplementary Figure S3. Full images of the blots from figure 3A.

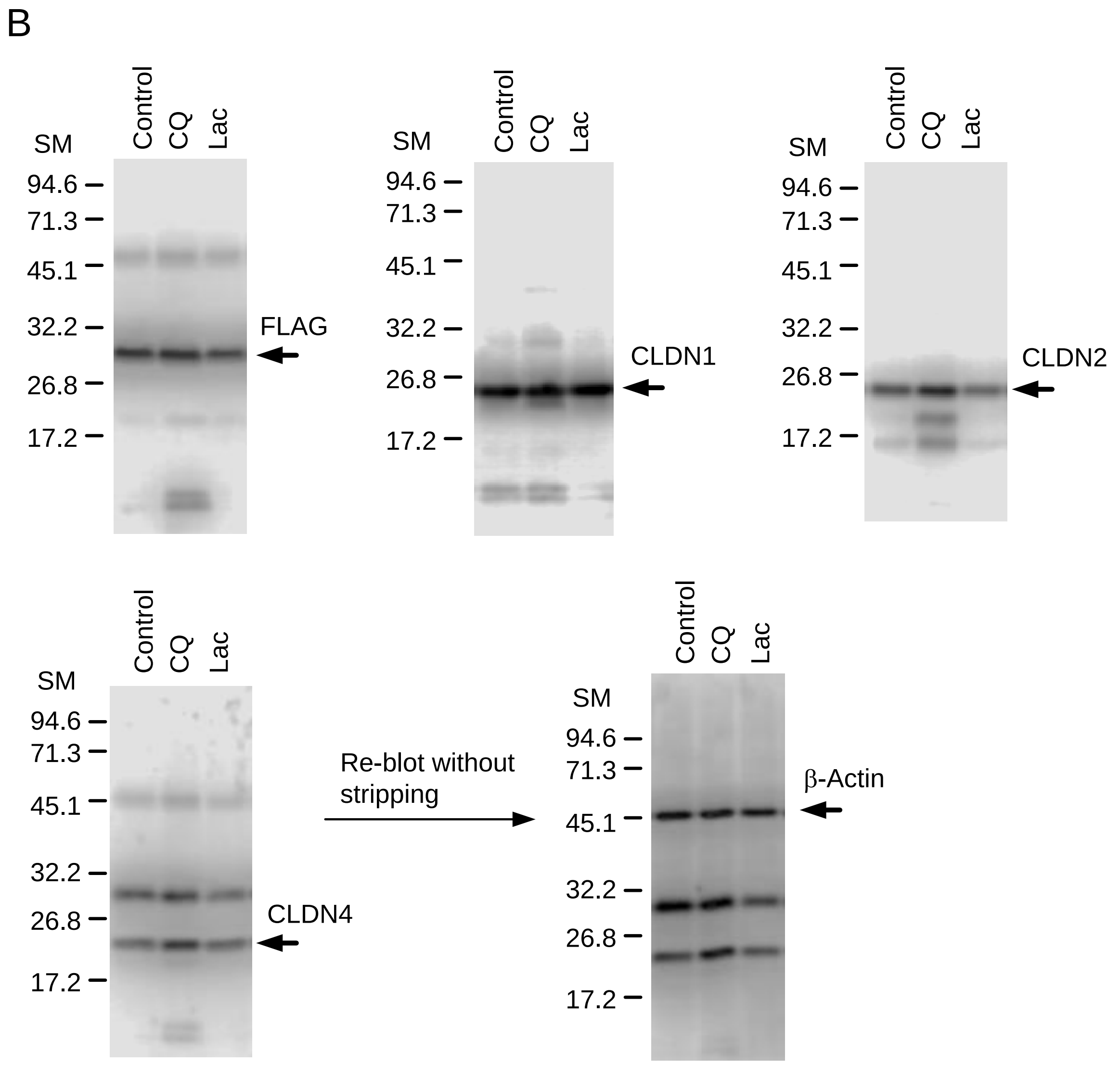

Supplementary Figure S4. Full images of the blots from figure 3B.

C

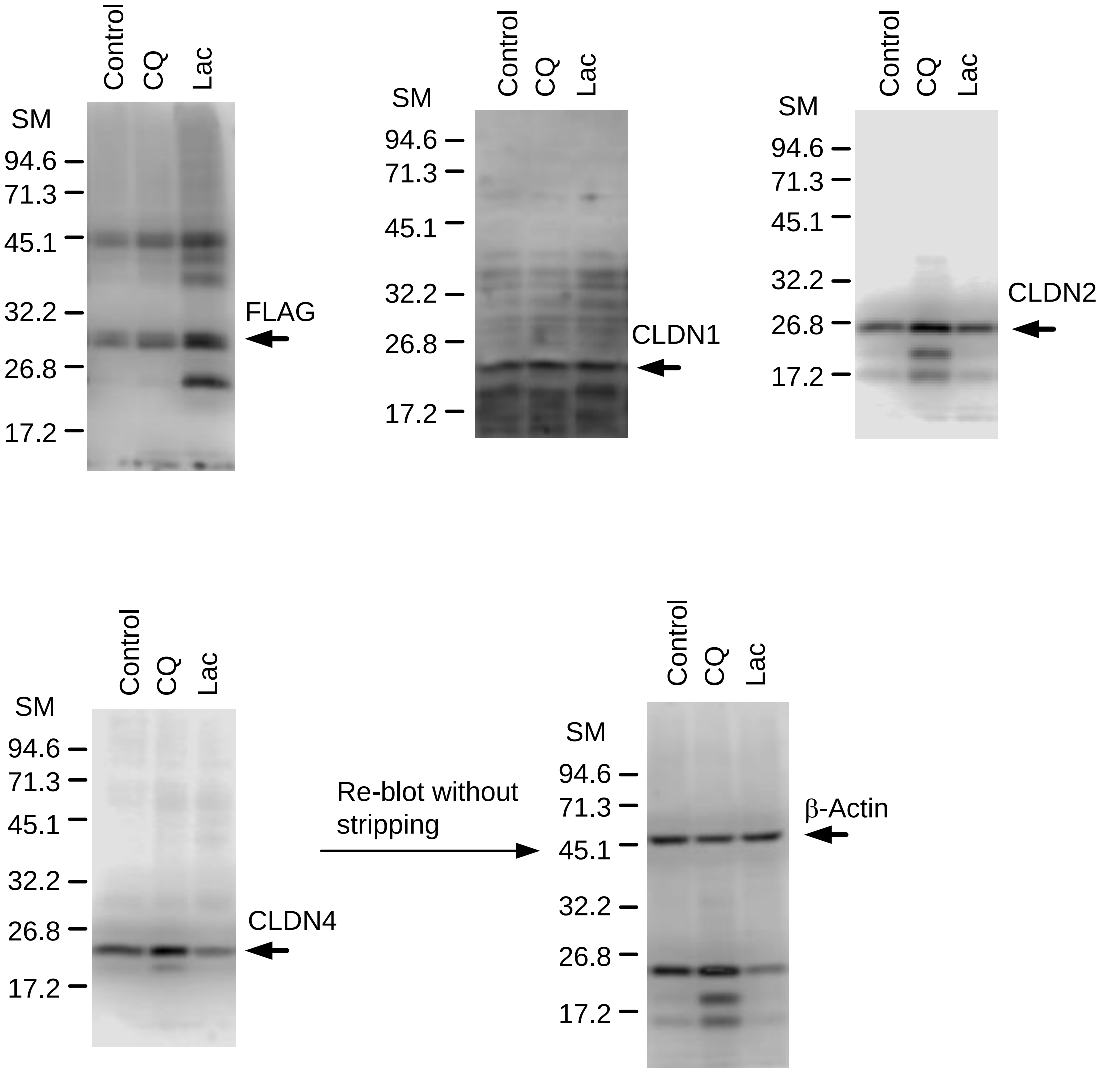

Supplementary Figure S5. Full images of the blots from figure 3C.

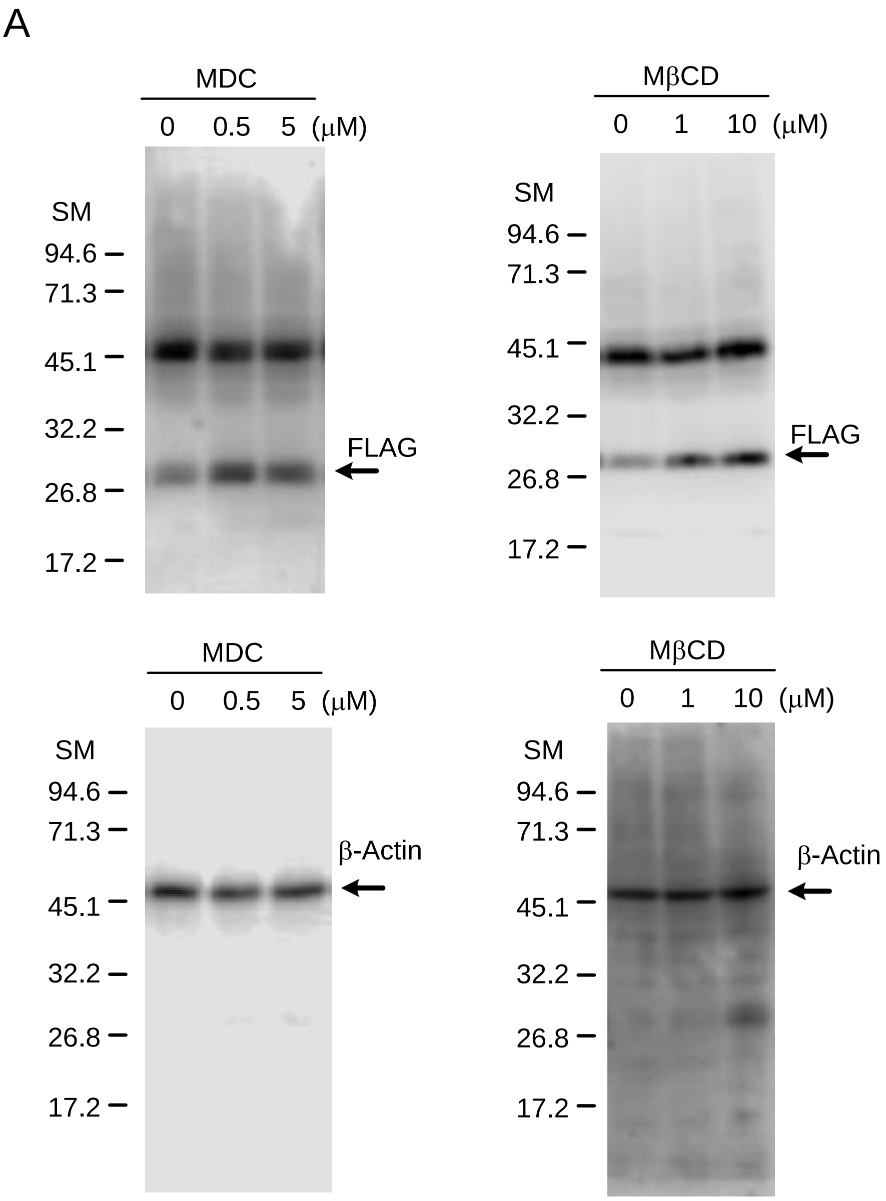

Supplementary Figure S6. Full images of the blots from figure 4A.

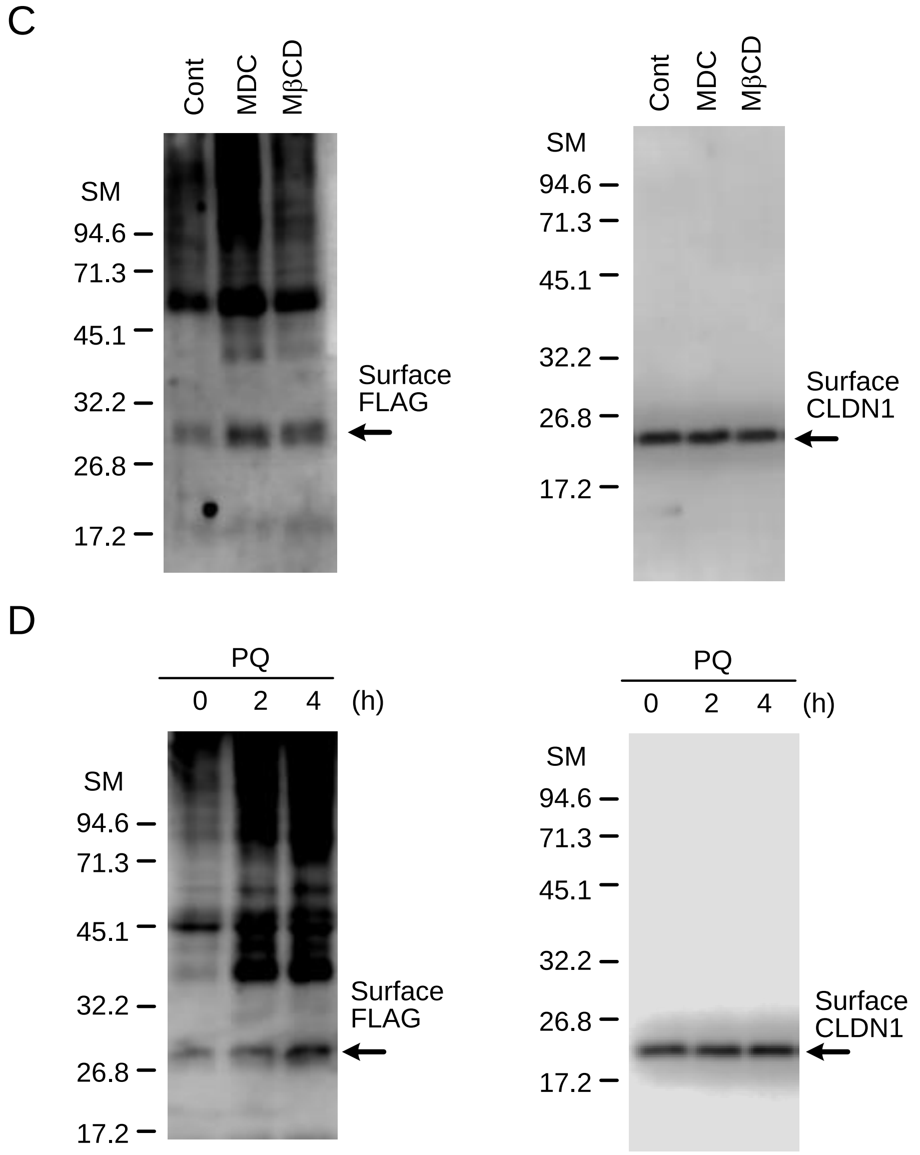

Supplementary Figure S7. Full images of the blots from figure 4C and 4D.

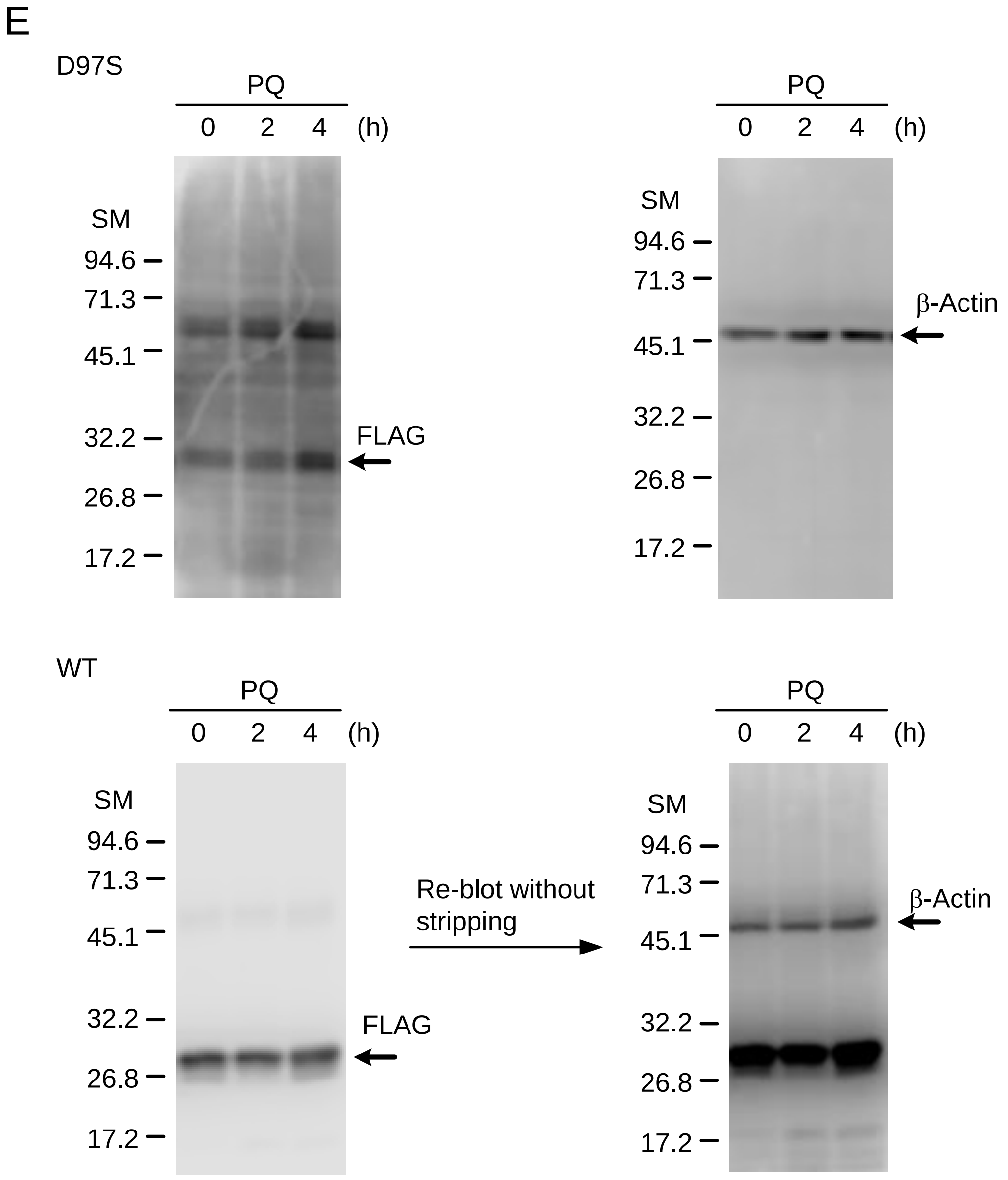

Supplementary Figure S8. Full images of the blots from figure 4E.

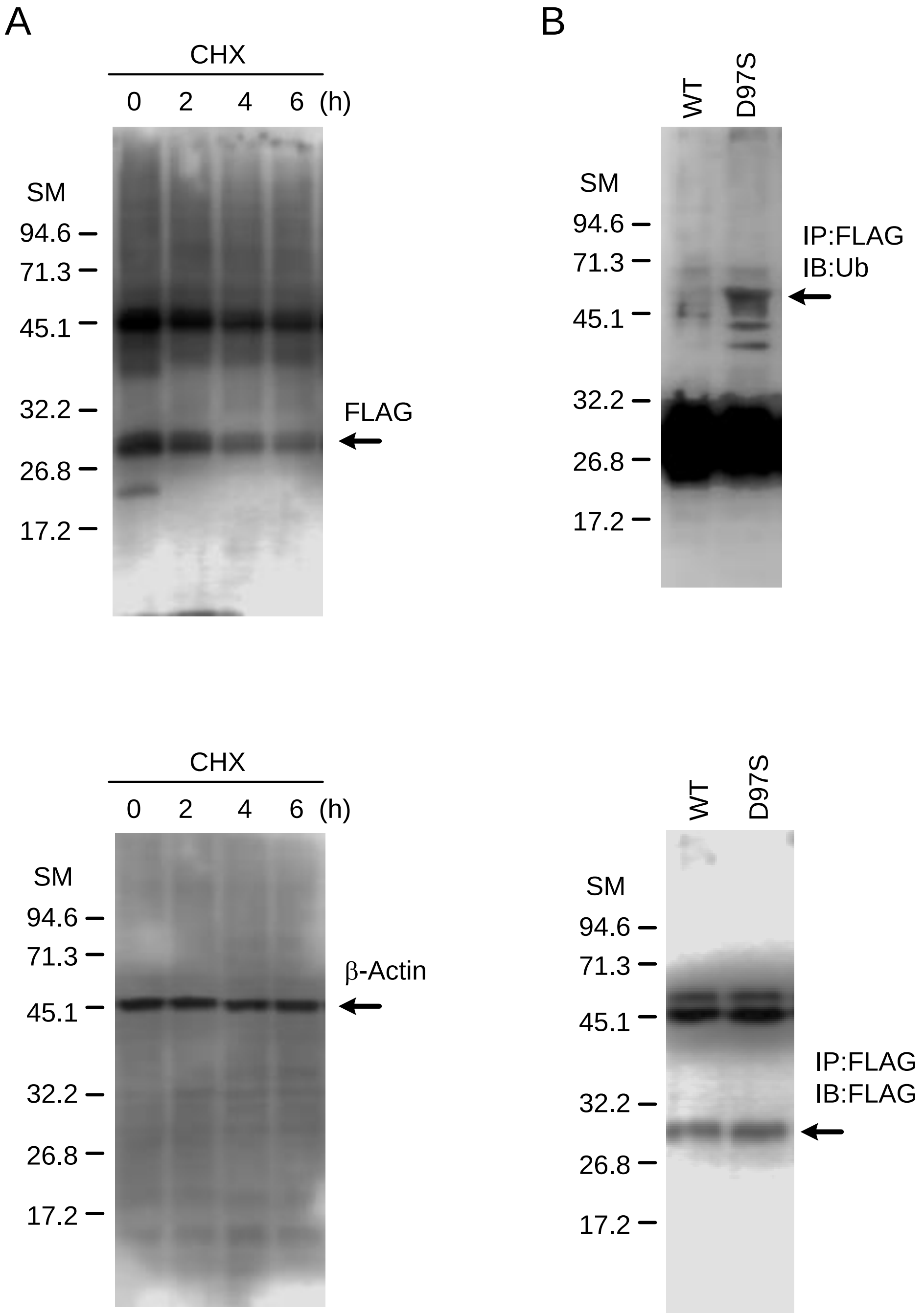

Supplementary Figure S9. Full images of the blots from figure 6A and 6B.

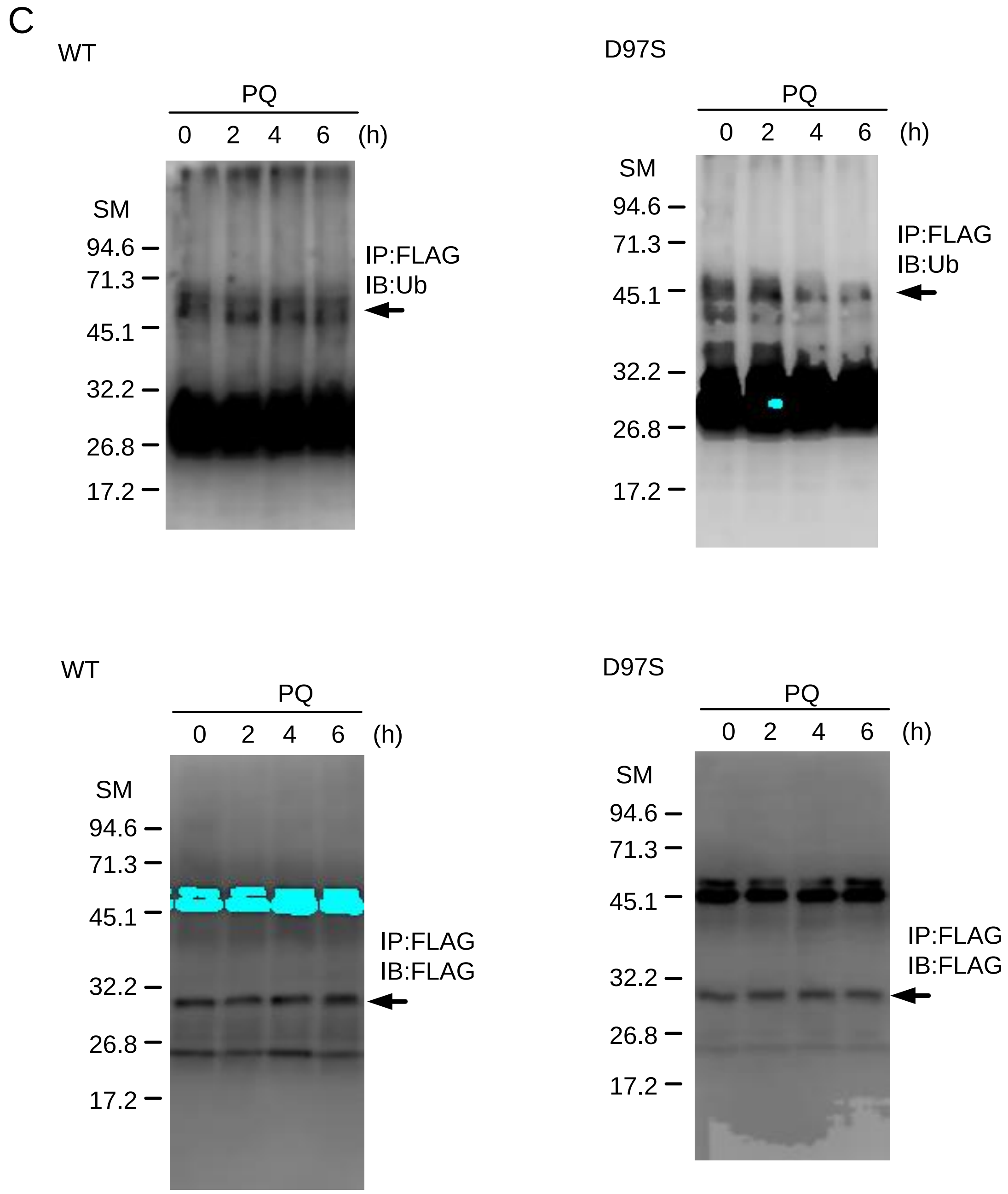

Supplementary Figure S10. Full images of the blots from figure 6C.

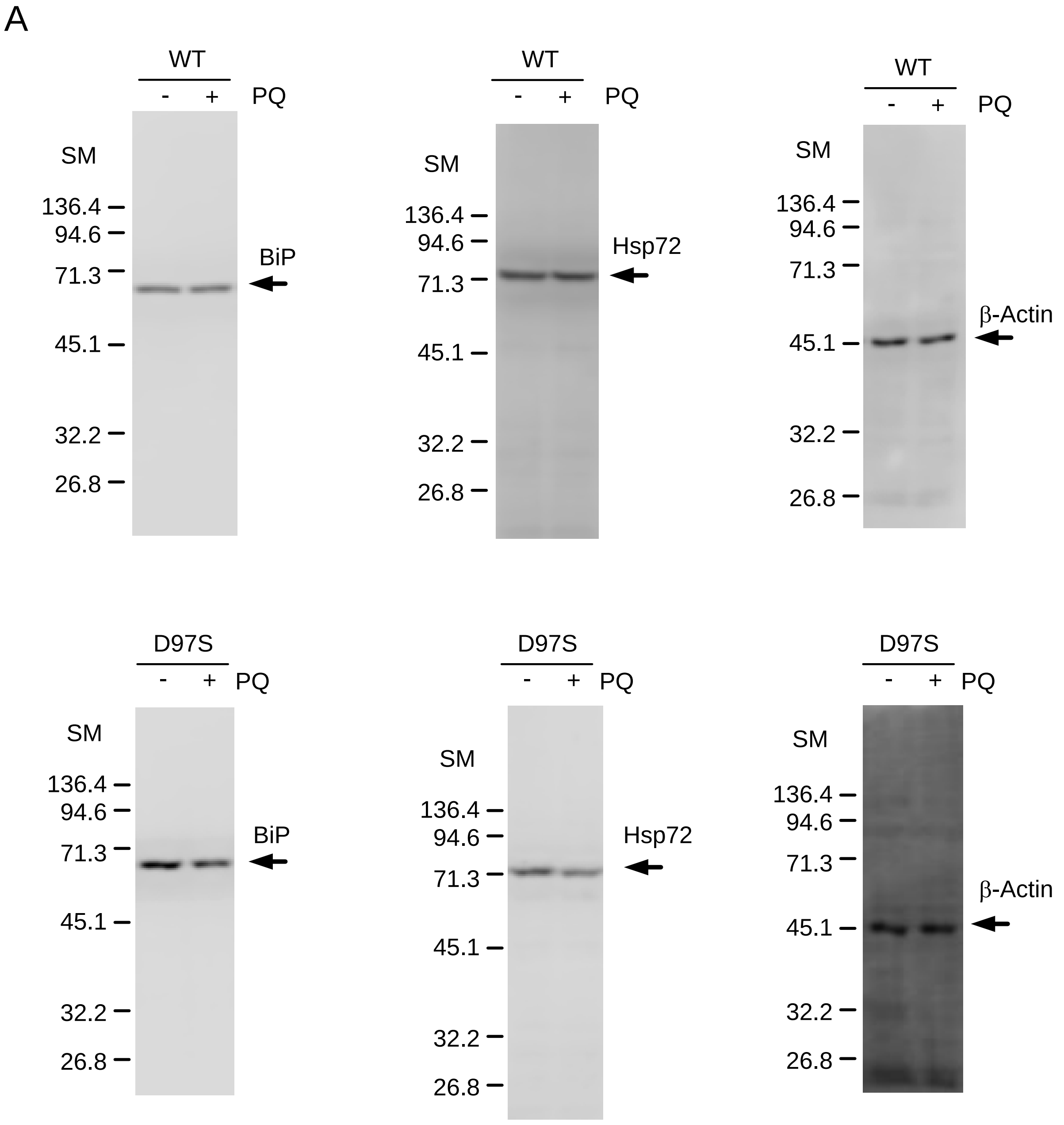

Supplementary Figure S11. Full images of the blots from figure 9A.

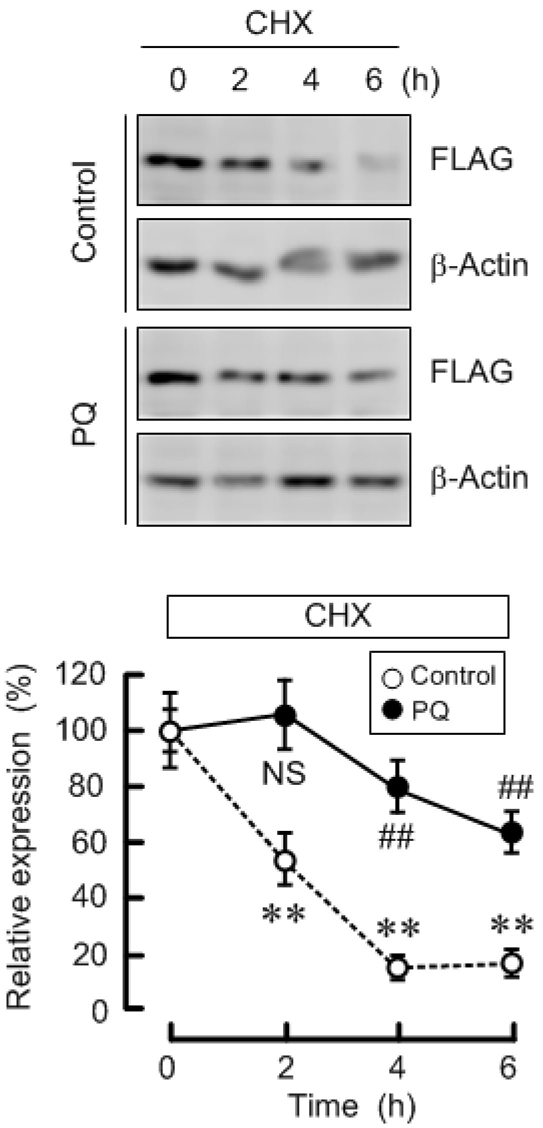

Supplementary figure S12. Effect of primaquine on protein stability of the R131C mutant. MDCK cells expressing the FLAG-tagged R131C mutant were treated with cycloheximide (CHX, 5 μM) and primaquine (PQ, 100 μM) for the indicated periods. After collecting cell lysates, the aliquots were blotted with anti-FLAG and anti-β-actin antibodies. The band densities of proteins are represented as the percentage of the values at 0 h. The full-length blot images are shown in Supplementary Figures S13. n = 3 in three independent experiments. ** $P < 0.01$ compared with 0 h. ## $P < 0.01$ and NS $P > 0.05$ compared with -PQ.

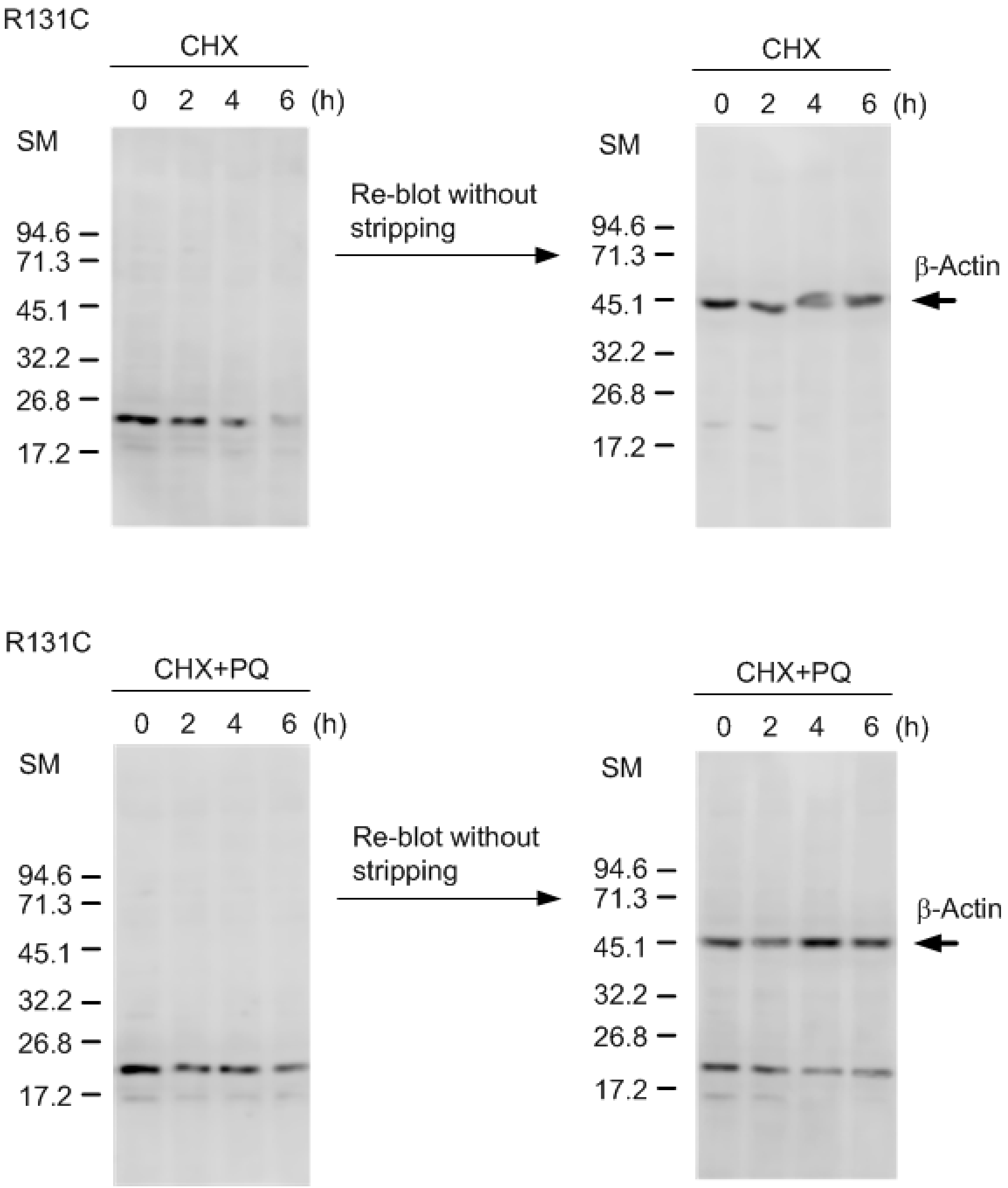

Supplementary Figure S13. Full images of the blots from supplementary figure S13A.

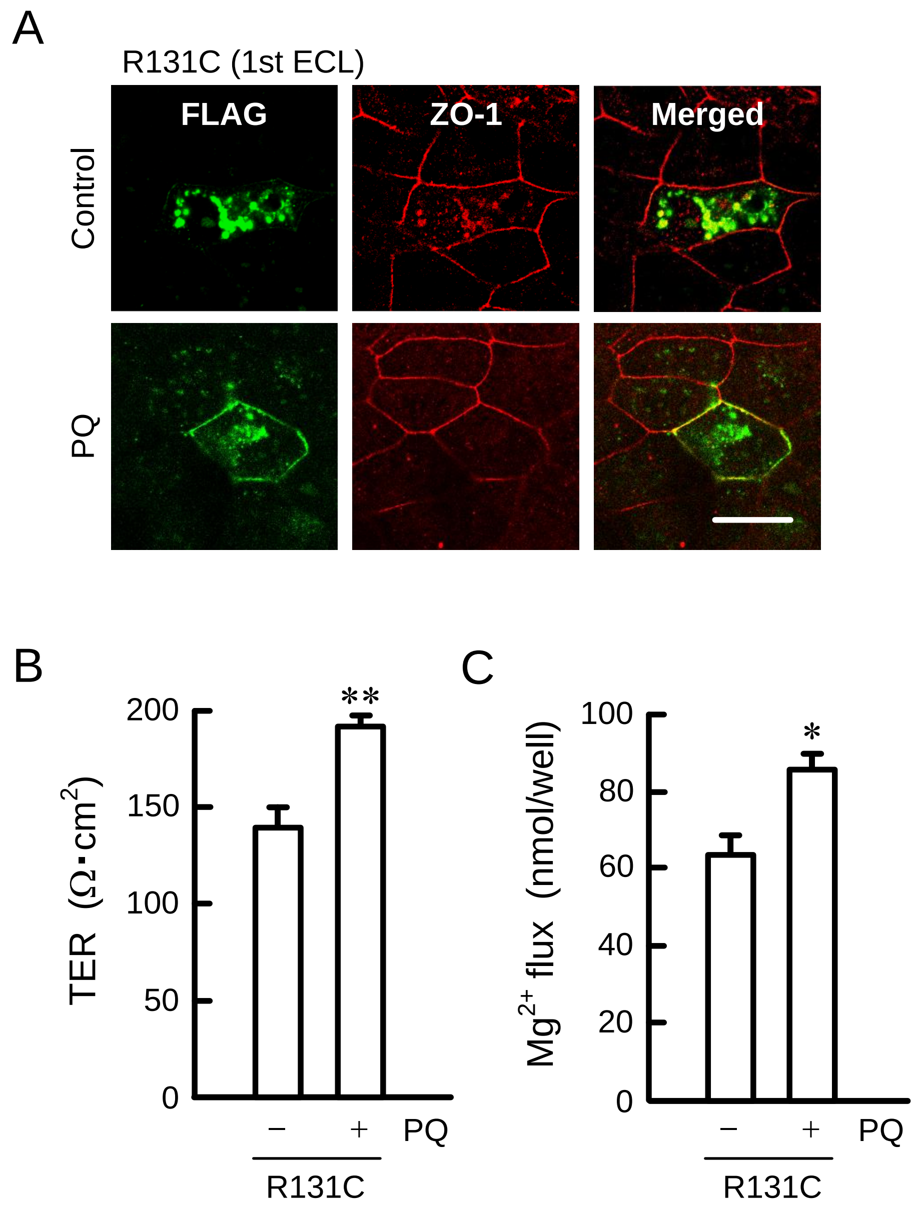

Supplementary figure S14. Effects of primaquine on the subcellular localization of R131C mutant and paracellular permeability. MDCK cells were transiently transfected with FLAG-tagged R131C mutant. The cells were incubated for 2 h in the absence or presence of 100 μM primaquine (PQ), and then stained with anti-FLAG (CLDN16) and anti-ZO-1 antibodies. Merged images are shown on the right. The scale bar indicates 10 μm. (B and C) TER was measured using a volt ohmmeter. Transepithelial Mg^{2+} permeability from the apical to basal compartment was measured using XB-1. n = 4 in four independent experiments. ** $P < 0.01$ and * $P < 0.05$ compared without -PQ.

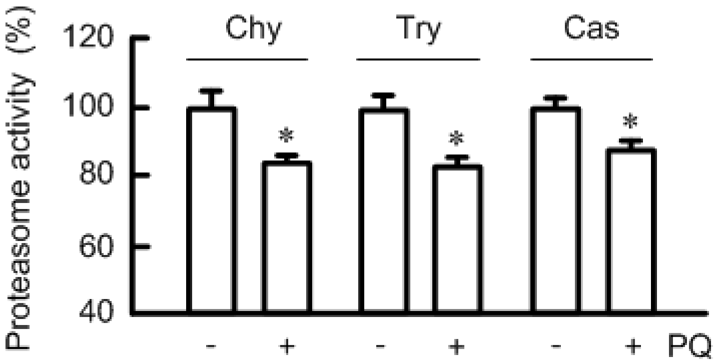

Supplementary figure S15. R131C mutant-expressing cells were incubated in the absence and presence of primaquine (PQ, 100 μM) for 6 h. After collecting the cytosolic fraction using a passive buffer, chymotrypsin-like (Chy), trypsin-like (Try), and caspase-like (Cas) proteasome activities were measured using each selective substrates. n = 4 in four independent experiments. * $P < 0.05$ compared with -PQ.
